# Provider perceptions of the anticipated benefits, barriers, and facilitators associated with implementing a stepped care model for the delivery of addiction and mental health services in New Brunswick: a mixed-methods observational implementation study

**DOI:** 10.1186/s13033-023-00611-9

**Published:** 2023-11-15

**Authors:** Alesha King, Laura M. Harris-Lane, Stéphane Bérubé, Katie Burke, AnnMarie Churchill, Peter Cornish, Bernard Goguen, Alexia Jaouich, Joshua A. Rash

**Affiliations:** 1 Department of Psychology, Memorial University of Newfoundland, 230 Elizabeth Ave, St. John’s, NL A1B 3X9 Canada; 2 Addiction & Mental Health Services, Department of Health, , Government of New Brunswick, Fredericton, Canada; 3 Stepped Care Solutions, Mount Pearl, Canada; 4 Counseling and Psychological Services, University of California Berkeley, Berkeley, USA

**Keywords:** Stepped mental healthcare, Stepped Care 2.0, One-at-a-Time therapy, Implementation science, Mental health, Benefits and barriers

## Abstract

**Background:**

Providers who work within addiction and mental health (A&MH) services in New Brunswick (NB), Canada completed training in Stepped Care 2.0 and One-at-a-Time (OAAT) therapy as part of a provincial practice change initiative to implement a provincial stepped care model. The present study aimed to identify: (1) the perceived acceptability and feasibility of the SC2.0 model; (2) the perceived benefits, barriers, and facilitators to implement SC2.0 in practice; and (3) perceived impacts on clinical practice.

**Methods:**

This is a mixed-methods observational implementation study. Quantitative surveys were completed after training courses. Open-ended responses were collected after completion of SC2.0 training. A subset of providers who completed surveys were asked to participate in semi-structured interviews. Descriptive statistics were used to describe results from surveys. Open-ended responses and semi-structured interviews were compiled and thematically synthesized in an iterative process using a grounded theory framework. Quantitative and qualitative data were triangulated to build an in-depth understanding of provider perceptions.

**Results:**

316 providers completed surveys and responded to open-ended prompts. Interviews were completed with 28 of those providers. SC2.0 was deemed to be acceptable, a suitable fit, and feasible to implement. Perceived benefits included: (1) timely access to services; (2) increased practice efficiency; and (3) increased availability of services. Perceived barriers included: (1) insufficient availability of resources to populate a SC2.0 continuum of care; (2) provider complacency with their current practice; and (3) difficulty for clients to accept and adjust to change.

**Conclusions:**

Identifying the perceived benefits, facilitators, and barriers to adopting stepped care in practice can lead to targeted implementation strategies and the collection of data that can inform continuous improvement cycles.

## Introduction

**Fig. 1:**
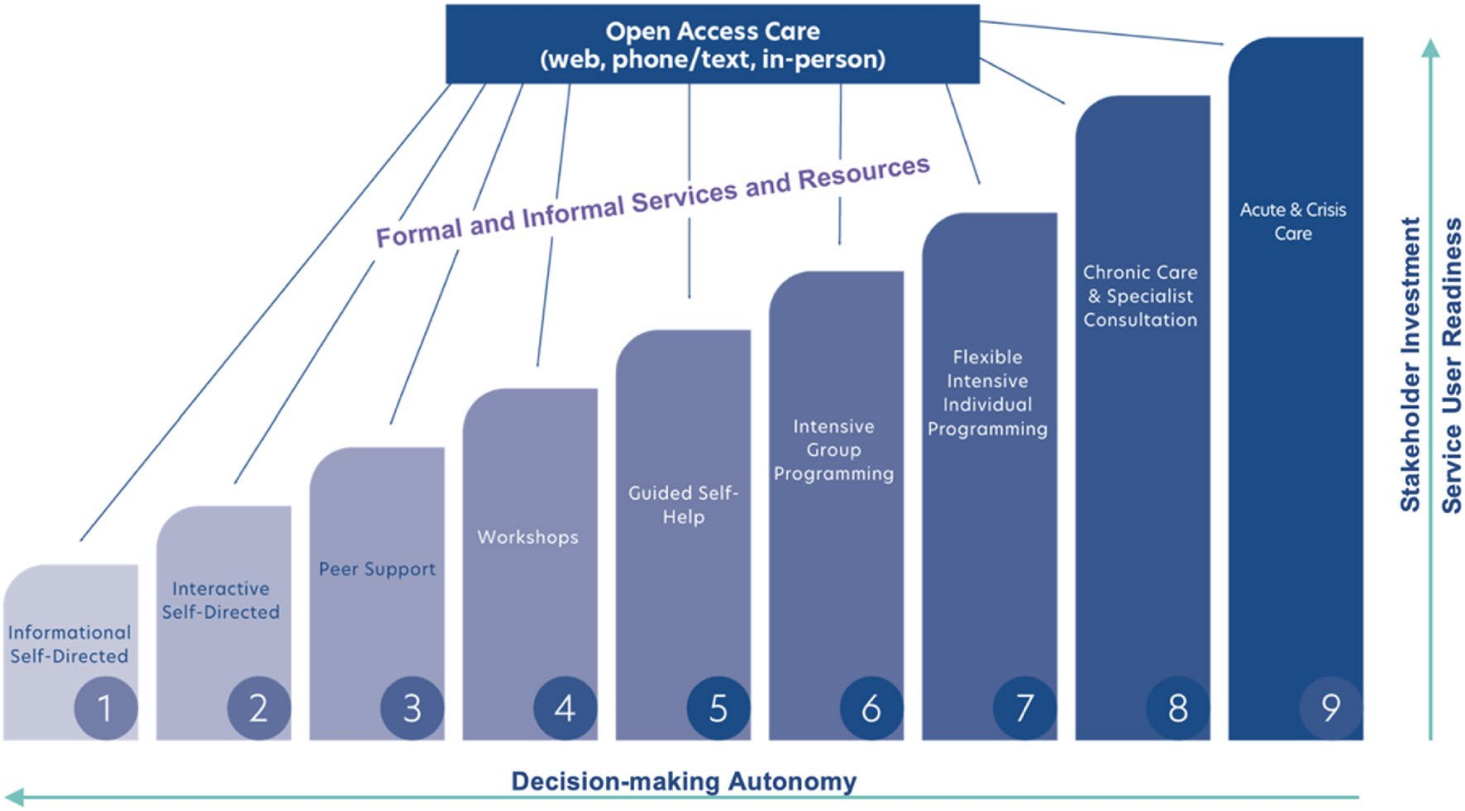
The Stepped Care 2.0 Intervention Steps. A visual representation of the dimensions and intervention intensities of Stepped Care 2.0. As treatment intensity increases with each step, stakeholder investment and readiness increase while autonomy decreases [2]

Adapted from the United Kingdom Stepped Care (SC) model [1], Stepped Care 2.0 (SC2.0) is a model of stepped mental healthcare that seeks to provide clients with the right care at the right time, while allowing same-day services [2]. In this model, clients are directly involved in their recovery, and can avail of care at different intensities that can be matched to their needs, preferences and readiness to engage [2, 3], refer to Fig. 1. Through a collaborative approach, clients and providers develop a care plan based on the client’s readiness (i.e., willingness and capacity to engage) and autonomy, as well as the required stakeholder investment at community, organization, and system levels [2]. Care plans can be adjusted as needed based on results from continuous outcome monitoring, and clients preferences and needs [2].

**Fig. 2:**
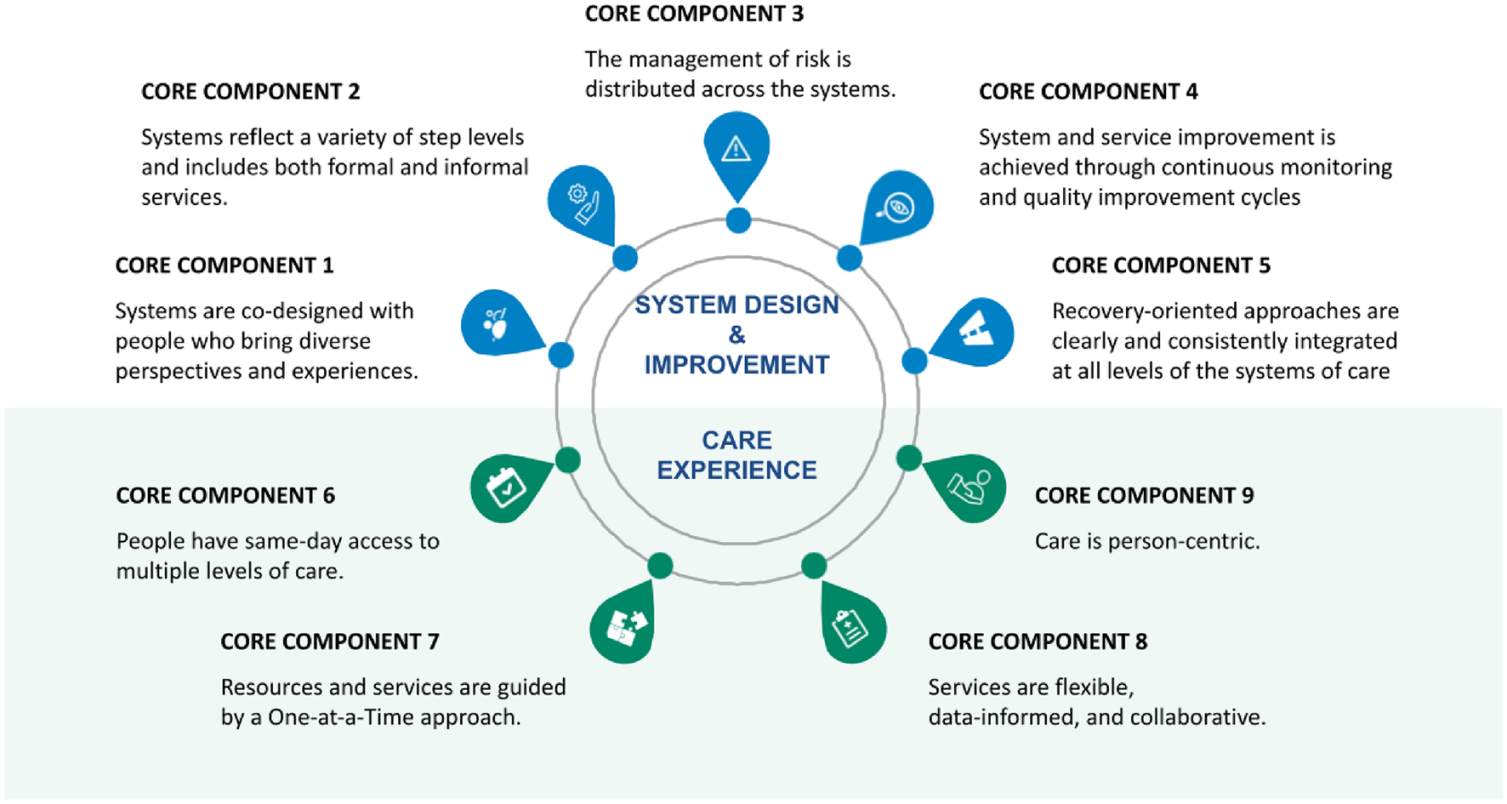
Core Components of Stepped Care 2.0. Diagram of the 9 Core Components of Stepped Care 2.0 and their description [4]

There are 9 core components of the SC2.0 model, five focused on system design and improvement, and four focused on the client care experience, refer to Fig. 2 [2]. Pertaining to system design and improvement, the system should: (1) be designed by individuals of diverse experiences and perspectives (e.g., leadership, service providers and service users, internal staff, community members); (2) reflect various step levels which include formal and informal services; (3) distribute the management of risk throughout the system; (4) undergo continuous improvement cycles based on key performance indicators to allow for an evolving system that is always well situated to provide quality care; and (5) integrate clear and consistent recovery principles at all levels within the system. Core components related to the client care experience include: (1) capacity for same-day access to services at multiple levels of care intensity; (2) each encounter is treated as a stand-alone and helpful interaction aimed at addressing the client’s top of mind concern; (3) care is flexible, data informed and collaborative; and (4) care is person-centric, considering client readiness and preferences.

The evidence supporting the SC2.0 model is promising at best given variability in the evidence supporting each core component of the SC2.0 model. For example, reviews attest to the benefit of measurement-based care for the management of addiction and mental health (A&MH) concerns, though risk of bias among included studies is not always evaluated [5–7]. Similarly, a body of evidence is emerging which attests to the benefits of addressing the client’s top of mind concern through the use of one-at-a-time (OAAT) therapy (commonly referred to as single-session therapy) [8–11], though it is not always clear that the intervention was delivered as intended or which concerns can be effectively addressed. Evidence for certain core components do not come from rigorous trials, but rather best practice guidelines. For example, integration of clear and consistent recovery principles within the delivery of A&MH services represents a best practice recommended by the Mental Health Commission of Canada [12]. The best practice guidelines on recovery-oriented practice were informed by empirical (e.g., systematic reviews) and grey (e.g., policy documents, international guidelines) literature, and books written by field experts, and were developed in consultation with leaders, experts, recovery champions, and people with lived and living experience. While best practice guidelines were informed by strong evidence (i.e., meta-analyses and systematic reviews), within the pyramid of evidence strength, it is clear that more rigorous research is needed on this SC2.0 core components.

Clinical practice guidelines for the management of common mental health concerns often recommend stepped care models to optimize resources and increase access [13–15]. These models of care are typically classified as stratified (i.e., clients’ treatment intensity is assigned according to symptom severity) or progressive (i.e., most client’s receive lower-intensity treatment despite symptom severity) [16]. Stepped care models have been shown to have positive effects on rates of recovery for common mental health disorders, such as depression and anxiety [17–19]. An observational cohort study in the UK that analyzed retrospective data (*n* = 16,723) over a 4-year period found that patients in a progressive treatment stepped care context were 1.5 times more likely to reach recovery than those patients in a stratified stepped care context [16]. Similarly, systematic reviews suggest that stepped care models can result in improvement in the treatment of anxious [20] and depressed mood [21], as well as reducing substance use [22]. It is difficult to ascertain the true effectiveness of stepped care models for the management of A&MH concerns due to variability in definition of stepped care models, implementation, and outcome measures [23].

The SC2.0 model is unique when compared to stratified and progressive models in that treatment is flexible and collaborative, and can begin at any level in consideration of the clients needs, preferences and readiness [2]. While risk of bias is high (i.e., due to insufficient consideration of confounding factors, sampling bias, lack of a-priori power calculation, measurement bias, and reporting bias, etc.) [24], preliminary evidence suggests that implementation of the SC2.0 approach can contribute to significant reductions in wait times for care. As evidenced in Newfoundland and Labrador (NL) and Northwest Territories (NWT), wait times for Addictions and Mental Health (A&MH) services decreased by 68% and 79%, respectively, following the implementation of a provincial SC approach [3, 24]. This may be, in part, due to the systematic distribution of appropriate resources [2], relieving stress that is often placed on high-intensity interventions.

While research has highlighted the outcomes of SC and SC2.0 approaches for clients and A&MH systems, little research has explored the implementation process, including barriers and facilitators to effectively enacting such models of care.

### One-at-a-time therapy

One characteristic that differentiates SC2.0 from other SC models is the incorporation of single-session thinking into access points, through OAAT therapy (core component #7 in Fig. 2). Using an OAAT therapeutic approach, each interaction with a client is viewed as a stand-alone and helpful interaction aimed at addressing the client’s top of mind concern. Providers work with clients, leveraging existing skills, strengths, and connections to address one top of mind concern of the client in one session [25]. This method presumes that a single session may be all that a client needs or is willing to engage with, but does not preclude future sessions. The client also has autonomy to return as needed.

Key principles of OAAT therapy include emphasizing the clients’ abilities and strengths rather than pathology, and the belief that big problems do not always require big solutions. An OAAT therapy approach also recognizes that some clients are looking for pragmatic help, and that a series of small changes can produce a meaningful impact. OAAT therapy integrates well with the SC2.0 model, as it facilitates rapid access to client-centric care by focusing on the client's top-of-mind concern in that session, and is often used as an entry point to the SC2.0 model (e.g., Doorways drop-in mental health clinics in NL) [3]. While usually incorporated as an entry point to care, OAAT therapy is applicable across a continuum of care, from low-intensity to high-intensity services.

Previous research has highlighted the utility of OAAT therapy in treating clients’ top-of-mind concerns and increasing accessibility to services through the drop-in approach [26]. For example, an evaluation of 524 clients from two family counseling agencies reported that clients who received single-session therapy by walk-in demonstrated a faster rate of improvement and were less distressed by 4-week follow-up than those who received traditional counselling with a wait-list [27]. Similarly, OAAT therapy has demonstrated promising effects for various populations (e.g., children, youth, and adults) and presenting concerns (e.g., anxiety disorders, conduct problems, psychological distress) [8, 10, 28, 29].

### New Brunswick initiative

The province of New Brunswick (NB) is situated on the east coast of Canada and has a population of over 800,000 individuals [30]. Addiction and mental health services are provided through a partnership between the Department of Health (DoH) and two Regional Health Authorities: Vitalité Health Network and Horizon Health Network. While there is a need for additional higher intensity services for both adult and children and youth who experience more severe substance use and mental health challenges (e.g., schizophrenia, complex trauma and neurodevelopmental issues), the most frequent reasons for referral to community services are issues related to anxiety and depression. Individuals with these issues also tend to wait the longest for services. The New Brunswick A&MH Services offers a broad continuum of care, but there are some gaps, including: (1) the number of treatment beds for concurrent substance use and mental health disorders; (2) services for individuals with neurodevelopmental issues; and (3) supportive housing services for individuals with A&MH concerns [31, 32]. While creating additional treatment beds and facilities does have its challenges, probably the biggest obstacle to overcome will be that of limited human resources. This includes direct service providers and system planners.

To combat protracted wait times and improve access to services across the A&MH system, the province of New Brunswick (NB) is implementing a 5-year phased approach to enact a provincial SC2.0 model to accomplish the following objectives: (1) improve population health; (2) apply early intervention; (3) match individuals to effective care; (4) improve access to services; and (5) reduce drug-related impacts [31]. While rigorous evidence from studies at low risk of bias is currently lacking, the province proceeded to implement OAAT therapy within a provincial SC2.0 framework given that: (1) core components of the SC2.0 model are supported by evidence or best practice statements, including the implementation of OAAT therapy for A&MH concerns; (2) reductions in wait times have been observed among provinces that have implemented OAAT therapy within and SC2.0 context; (3) lack of access to timely and integrated MH&A services in NB necessitated prompt action, and evidence for SC2.0 to effectively combat these concerns was promising; and (4) this project proceeded within an evaluation framework that offered insights into change observed over time. As a first step, providers working in A&MH services within the two regional health authorities, Vitalité and Horizon Health Networks, and provincial school districts underwent training in SC2.0 and OAAT therapy as part of a provincial practice change initiative that commenced in July 2021. Training involved the completion of two asynchronous courses: OAAT therapy and SC2.0 that were developed by Stepped Care Solutions, a Canadian not-for-profit consultancy group and the developers of the SC2.0 model. These courses were designed to develop the knowledge and understanding of OAAT therapy and the SC2.0 model by providing examples of how its principles are applied. This is done through text and video explanations, sample interactions with clients, and checkpoints for participants to complete.

In addition to the development of a 5-year action plan, the province of NB has partnered with Memorial University of Newfoundland to monitor implementation outcomes and facilitate continuous improvement cycles within the system. This includes the exploration of the acceptability, feasibility, and utility of SC2.0 model, and the key barriers and facilitators to an effective and successful implementation into practice. Information gathered from providers completing the two asynchronous courses and associated evaluation offers important insights for the provincial implementation team to foster growth and improvement as the A&MH system moves towards achieving quality, equitable, and sustainable care. To date, the province has implemented OAAT therapy into practice, and is currently working to connect the provincial continuum of care in alignment with a provincial SC2.0 model.

## Present study

We conducted a mixed-methods observational implementation study to gain a better understanding of providers’ perceived acceptability and feasibility of the SC2.0 model, as well as perceived benefits and barriers of implementing SC2.0 and OAAT therapy into practice. Information was collected through: (1) administration of surveys that contained measures of acceptability and feasibility, and open-ended questions about barriers, facilitators and perceived benefits; and (2) conducting semi-structured interviews. The results of this investigation will provide insight to other organizations looking to enact large-scale implementations of SC models in A&MH systems.

## Methods

A mixed methods design was adopted that triangulated data from surveys with information obtained from qualitative interviews.

### Participants

#### Surveys

Providers (e.g., social workers, psychologists, nurses, counselors) working within A&MH Services in Vitalité and Horizon Health Networks, and the seven provincial school districts in NB were eligible to participate. Providers who work with adult populations primarily work in community A&MH clinics, while providers who work with child and youth populations work on integrated teams. The Integrated Service Delivery (ISD) model for children and youth offers comprehensive services (i.e., academic, addiction and mental health, family relationships, and physical health and wellness through collaboration between providers who work within the health networks and school districts, and in conjunction with the Department of Social Development and the Department of Justice and Public Safety.

Providers were recruited through emails and virtual meetings with the provincial working group of Adult and Child & Youth services leaders (i.e., managers, clinical leads, and directors). Providers were aware that participation in research was an optional component of the provincial change initiative. Gift-cards valued at $20 were distributed at three timepoints (a total value of $60 per participant) as an incentive to participate. This study was approved by the NL Health Research Ethics Board (Ref# 2021.094), Horizon Health Network Research Ethics Board (Ref# 2021-3015), and the Vitalité Health Network Ethics Office (Ref# 2957). Consent to participate was obtained electronically.

#### Interviews

Providers who completed asynchronous courses and consented to be contacted about additional research opportunities were contacted through e-mail and invited to participate in a qualitative interview. Purposive sampling was used to ensure equal representation of providers who practiced with adult and child/youth populations. Participants completed an interview on SC2.0 or OAAT therapy, and were offered one $20 gift card upon commencing an interview as an incentive for participation. Protocols for qualitative interviews were approved by the NL Health Research Ethics Board (Ref# 2021.178).

### Procedures

#### Surveys

Providers who consented to participate in research were invited to complete study questionnaires through the survey platform Qualtrics, and the SC2.0 and OAAT therapy courses, in a set order. Providers who did not consent to participate in the optional research received immediate access to the courses. Adult providers who consented to participate completed the OAAT therapy course followed by the SC2.0 course, while child and youth providers completed the SC2.0 course followed by the OAAT therapy course. As outlined in Table 1, providers completed questionnaires before and after the OAAT therapy and SC2.0 courses. Questionnaires and courses were made available in English and French.

**Table 1:** Order of assessments and courses

Order of assessments and courses—adult providers					
Measure	T1	OAAT therapy course	T2	SC2.0 course	T3
Demographics	X				
Acceptability, appropriateness, and feasibility					X
Readiness for organizational change-25					X
Commitment to change-18					X
Implementing stepped care in your practice					X

Order of assessments and courses—child and youth providers					
Measure	T1	SC2.0 course	T2	OAAT therapy course	T3
Demographics	X				
Acceptability, appropriateness, and feasibility			X		
Readiness for organizational change-25					X
Commitment to change-18					X
Implementing stepped care in your practice			X		

#### Interviews

Interviews were conducted using the video-conferencing platform Zoom [33] and spanned 60 min. Interviews were recorded and transcribed verbatim, thematically analyzed, and synthesized in an iterative process throughout data collection.

### Measures

#### Surveys

**Demographics** A demographics questionnaire was created to characterize the sample. Variables assessed included: practice setting, practice location, profession, years in practice, education, and primary population served.

**Acceptability, appropriateness and feasibility of intervention measure (AAFI)** was used to evaluate the acceptability, appropriateness, and feasibility of SC2.0 as a model of care delivery [34]. Sets of four items were used to measure each facet for a total of 12 items. Items were rated on a 5-point Likert scale, ranging from 1- “Completely disagree” to 5- “Completely agree”. The AAFI has demonstrated reliability, structural validity, and sensitivity to change [34]**.**

**Readiness for organizational change scale (ROCS-25)** assessed providers’ perceptions of their organization’s readiness to implement an SC2.0 approach [35]. Twenty-five items were scored on a 7-point Likert scale ranging from 1- “Strongly disagree” to 7- “Strongly agree”. Subscales of the measure included: (1) appropriateness; (2) management support; (3) change efficacy; and (4) personally beneficial. The ROCS-25 has demonstrated reliability and validity as a measure of factors that influence readiness [35].

**Commitment to organizational change (COC-18) questionnaire** evaluated providers’ commitment to organizational change, and specifically to changing the way they practice to align with SC2.0. Eighteen items were scored on a 7-point Likert scale ranging from 1- “Strongly disagree” to 7- “Strongly Agree”. Three subscales assessed affective commitment (i.e., desire to change), continuance (i.e., perceived cost associated with change), and normative commitment (i.e., perceived obligation to change) to changing their practice. The COC-18 has demonstrated reliability and validity in measuring commitment to change [36].

**Implementing stepped care in your practice (ISCP) open-ended questions** Providers responded to a series of open-ended questions about anticipated barriers and benefits that implementing SC2.0 in their practice would have on clients, clinical practice, and their organization.

#### Interviews

A semi-structured interview guide was created to assess providers’ beliefs about the OAAT therapy and SC2.0 trainings and experiences implementing course content into practice. The interview guide was constructed in accordance with the Theoretical Framework of Acceptability [37]. Interviews were conducted by research assistants (AK, AM, LHL, CF, NKV, JD, AH, SF, DB), the majority of whom had previous experience working within the healthcare system. Research assistants underwent training and were supervised by a registered Psychologist (JAR).

**Theoretical framework of acceptability** Acceptability of OAAT therapy and SC2.0 training courses were explored in alignment with the Theoretical Framework of Acceptability [37], which consists of seven component constructs: affective attitude, burden, ethicality, intervention coherence, opportunity cost, perceived effectiveness, and self-efficacy. These seven constructs work together to form a prospective, concurrent, or retrospective model of acceptability and have been helpful in identifying areas of improvement (e.g., intervention coherence) in programs [38].

### Analysis

#### Surveys

Descriptive statistics were conducted on quantitative variables using IBS SPSS Statistics v25 [39] to describe: (1) characteristics of the sample; (2) acceptability, feasibility, and utility of stepped care; and (3) readiness for and commitment to organizational change. Independent samples t-tests were performed to evaluate differences between providers who served adult populations and those who served child and youth populations. A critical α = 0.01 was adopted for testing statistical significance to compensate for inflation in familywise error when performing multiple tests. Given the relatively large sample size, differences were only interpreted if the magnitude of effect exceeded *d* = 0.42, a recommended cutoff for minimum practical significance [40]. Missing data were not imputed given that analysis focused on descriptive statistics.

Responses from open-ended questions were compiled and each question was thematically synthesized in an iterative process following Braun and Clarke [41] recommendations for thematic analysis. Coders also drew from standards recommended by DeCuir-Gunby et al. [42]. Data-driven codes emerged from repeated examination of the raw data. Reviewers familiarized themselves with the open-ended responses, created preliminary coding categories, identified common and recurring themes, and refined and named themes through consensus meetings before proceeding with substantive coding. A codebook was developed and agreed upon by three independent coders (LHL, AK and AM) who met until consensus was achieved. Coding and synthesis of open-ended responses was completed in duplicate by two independent reviewers. Responses were organized by overarching theme, code, subcode and definition.

#### Interviews

Thematic analysis was used to organize and categorize patterns within data, using an inductive, constant comparison, descriptive approach [41]. Pairs of reviewers read transcripts to familiarize themselves with the data, created codes, and noted patterns. Potential themes were discussed, and transcripts re-read to refine themes in an iterative process [42]. Final themes were named and agreed upon through consensus meetings with the study team (AK, AM, LHL, CF, NKV, JD, AH, SF, DB, JAR).

## Results

### Sample characteristics

Out of ~ 800 A&MH providers who completed training, the present study contains a total of 401 participants completed surveys (50% response rate). Data from 85 providers were excluded due to missing responses on the ISCP survey. Analysis included data from 316 providers working within A&MH Services in NB. Descriptive statistics are presented in Table 2. Over half of the providers were trained in social work (*n* = 180, 57.0%) and practiced in an urban setting (*n* = 174, 55.2%). Among providers working with adult populations (*n* = 141, 44.6%), the vast majority worked within a community A&MH clinic (*n* = 127, 90.1%). Additionally, child and youth providers (*n* = 175, 55.3%) reported working in healthcare (*n* = 129, 73.7%), and education (*n* = 45, 14.2%) on the child and youth ISD teams.

**Table 2:** Descriptive statistics and characteristics of the sample that completed surveys

	Respondents		Non-Respondents	
	*N*	%	*N*	%
**Population served**				
Adults	141	44.6	55	50.6
Children/youth	175	55.3	30	49.4
Organization				
Vitalité	120	38.0	21	24.7
Horizon	149	47.2	46	54.1
School district	44	13.9	18	21.2
Other	3	0.9		–
Practice location				
Rural	141	44.6	35	41.2
Urban	174	55.1	50	58.8
**Practice setting**				
All providers				
Primary care clinic	3	0.9	4	4.7
Community A&MH clinic	127	40.2	30	35.3
Child and youth team (healthcare)	129	40.8	21	24.7
Child and youth team (education)	45	14.2	18	21.2
Other	12	2.8	12	14.1
Adult providers				
Primary care clinic	3	2.1	4	9.3
Community A&MH clinic	127	90.1	30	69.8
Other	11	7.8	9	20.9
Child and youth providers				
Child and youth team (healthcare)	129	73.7	21	50.0
Child and youth team (education)	45	25.7	18	42.9
Other	1	0.6	3	7.1
**Level of education**				
Doctoral	5	1.6	3	3.5
Master’s	86	27.2	20	23.5
Baccalaureate	191	60.4	48	56.5
Professional certificate	23	7.3	12	14.1
Other	11	3.5	2	2.4
**Provider profession**				
Nursing	42	13.3	22	25.9
Psychology	28	8.9	8	9.4
Social work	180	57.0	42	49.4
Counselling	12	3.8	4	4.7
Business administration/admin	11	3.5	2	2.4
Education	10	3.2	3	3.5
Occupational therapy	16	5.1	1	1.2
Other	17	5.4	3	3.5
**Professional role**				
Provider	254	80.4	66	77.6
Manager	13	4.1	2	2.4
Provider/manager	25	7.9	10	11.8
Business admin/admin	9	2.8	2	2.4
Clinic coordinator	11	3.5	4	4.7
Other	4	1.3	1	1.2

A&MH,  Addictions and Mental Health; Respondents,  providers with complete data who were included in analyses of survey data (N = 316); Non-Respondents, providers excluded from analysis of surveys due to incomplete responses to short-answer questions pertaining to implementing stepped care in your practice (N = 85)

A subset of 28 providers participated in semi-structured interviews to better understand the perceived barriers, facilitators, and benefits of the implementation of SC2.0 (*N*_SC_ = 12) and OAAT therapy (*N*_OAAT_ = 16) in practice. The interview sample included representation of providers working in adult (*N*_SC_ = 6; *N*_OAAT_ = 12) and child/youth (*N*_SC_ = 6; *N*_OAAT_ = 4) services.

### Acceptability, feasibility and organizational commitment to implementing stepped care 2.0

Descriptive statistics pertaining to perceived acceptability, feasibility, and organizational commitment to change can be located in Table 3. Results are depicted for the overall sample, and by population served. Providers were accepting of the implementation of SC2.0 in their practice (AAFI_Acceptability_; *M* = 4.28, *SD* = 0.64), believed that the model was a suitable fit (AAFI_Appropriateness_; *M* = 4.16, *SD* = 0.67), and was feasible to implement (AAFI_Feasibility_; *M* = 3.91, *SD* = 0.74). Providers also agreed that the organization could benefit from implementation (ROCS_Total_; *M* = 5.57, *SD* = 0.80) and there were rational reasons to implement SC2.0 (ROCS_Appropriateness_; *M* = 5.77, *SD* = 0.92). Providers agreed that there was sufficient management support for the implementation of the model (ROCS_Management Support_; *M* = 5.50, *SD* = 1.10), were confident in their ability to adopt SC2.0 principles into practice (ROCS_Change Efficacy_; *M* = 5.22, *SD* = 1.00), and believed that SC2.0 can be personally beneficial (ROCS_Personally Beneficial_; *M* = 5.77, *SD* = 1.34). Moreover, providers believed there was value to implementing SC2.0 and that doing so would serve an important purpose (COC_Affective Commitment_; *M* = 5.97, *SD* = 0.98). Providers perceived few costs associated with implementation (COC_Continuance_; *M* = 3.23, *SD* = 1.32), and did not express pressure to implement SC2.0 out of an obligation to the organization (COC_Normative Commitment_; *M* = 4.62, *SD* = 1.01). Providers who work with adult populations perceived greater support for the implementation of SC2.0, and viewed the implementation as more appropriate and feasible, refer to Table 3.

**Table 3:** Summary of quantitative findings

Measure	Total *M *(*SD*)	Adults *M *(*SD*)	Child and youth *M *(*SD*)	Effect size of difference
**AAFI**				
Acceptability	4.28 (0.64)	4.41 (0.59)	4.18 (0.66)	0.37*
Appropriateness	4.16 (0.67)	4.32 (0.61)	4.02 (0.68)	0.46*
Feasibility	3.91 (0.74)	4.15 (0.70)	3.71 (0.72)	0.62*
**ROC-25**				
Appropriateness	5.77 (0.92)	5.99 (0.83)	5.59 (0.96)	0.45*
Management support	5.50 (1.10)	5.81 (1.03)	5.22 (1.08)	0.56*
Change efficiency	5.22 (1.00)	5.42 (1.02)	5.05 (0.95)	0.38*
Personally beneficial	5.77 (1.34)	5.89 (1.41)	5.66 (1.26)	0.17
Total	5.57 (0.80)	5.80 (0.76)	5.38 (0.78)	0.54*
**COC-18**				
Affective	5.97 (0.98)	6.12 (0.94)	5.84 (0.99)	0.29
Continuance	3.23 (1.32)	3.18 (1.24)	3.28 (1.40)	0.07
Normative commitment	4.62 (1.01)	4.70 (1.05)	4.54 (0.97)	0.16

AAFI, Acceptability, appropriateness, and feasibility of intervention; ROC-25, Readiness for organizational change-25; COC-18, Commitment to organizational change-18

*p < 0.01

### Perceived organizational barriers

**Fig. 3:**
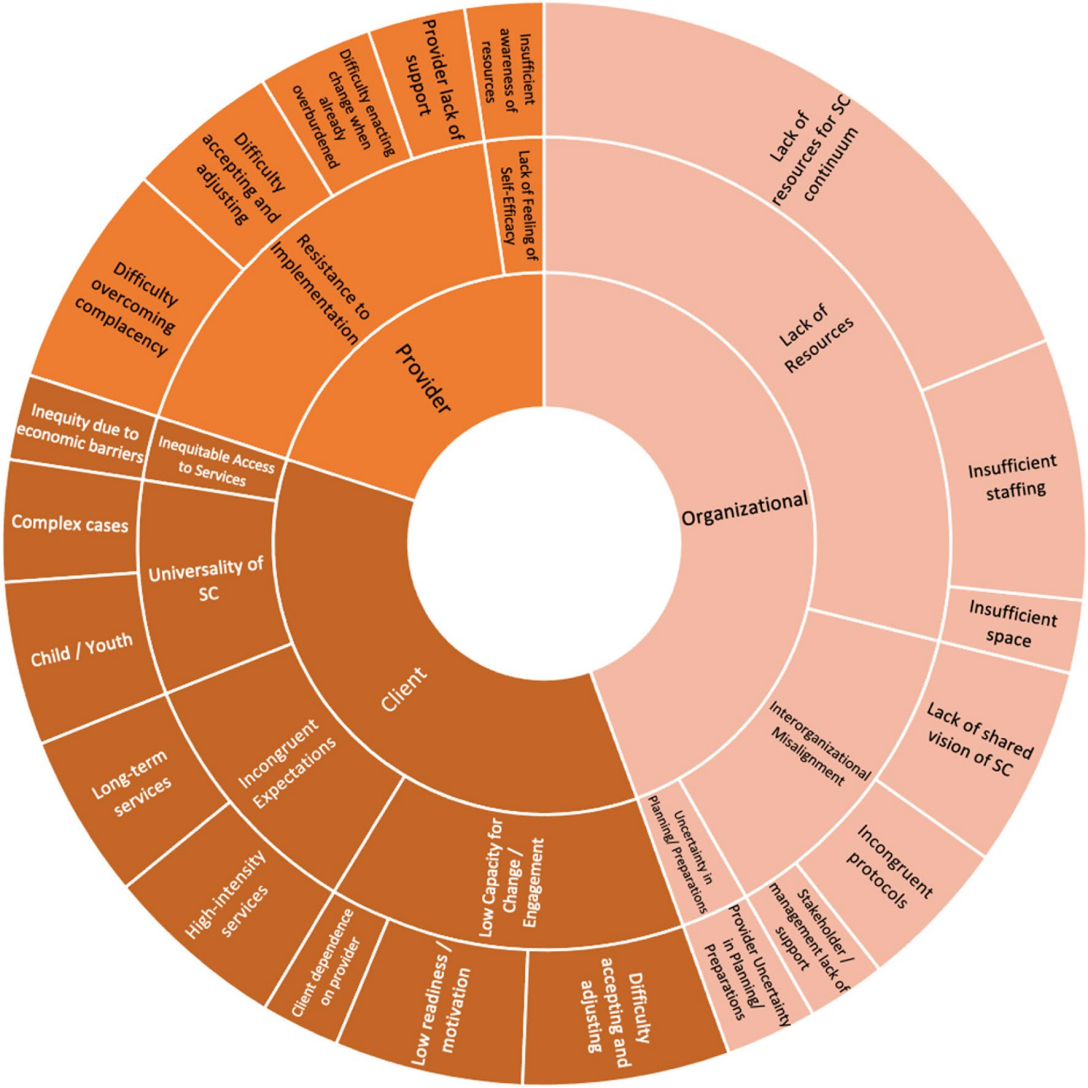
Summary of results for open-ended responses: perceived Barriers. Inner-most section represents the respective stakeholder the barrier would affect. The middle section represents the overarching theme, followed by the code in the outer-most section. Size of each box is determined by the number of provider responses per code

A summary of perceived barriers to implementing SC2.0 in practice are presented in Fig. 3. A complete description of each barrier and its associated SC2.0 Core Component are provided in Table 4. Open-ended responses (*N*_ISCP_ = 216) and interviews highlighted three themes: (1) insufficient resources to enact SC2.0; (2) interprofessional and interorganizational misalignment; and (3) uncertainty in organization planning.

**Table 4:** Summary of qualitative findings

Organizational barriers						
Theme	Description	OAAT Interview (*N* ≥ 3)	SC Interview (*N* ≥ 3)	ISCP (*N* ≥ 10)	Representative Quote	Associated SC2.0 core component
*Insufficient resources to enact SC2.0*						
**O1.1** Insufficient resources available for a SC2.0 continuum	Providers felt that there were insufficient resources to populate an SC2.0 continuum. This included resources for care (e.g., peer support, guided self-help, peer reviewed educational materials, etc.), and for increasing client literacy about the new model (e.g., handouts explaining the new care delivery model)	–	7	104	*I think it could work if we had the resources to be implemented and if we yeah, it would work if we had the resources, if we don't, then it's not going to work and I think you have to I have enough buy-in from people (P1074)*	CC2
**O1.1.1** Variance in available resources due to region	Different regions may have different resource availability, impacting providers’ ability to implement stepped care into their practice. Further, services may be inaccessible in rural communities due to distance and lack of transportation	–	3	–	*There's a lot of walk in counselling where they would just come to our office and meet with us but for us, like our office is at least 30 min from every school that we have cause our schools are in the outskirts. So they can't come to our office. Our communities are very small with not a lot of people that have methods of transportation to our offices (P1133)*	CC2
**O1.2** Insufficient space to enact OAAT therapy sessions within child and youth services	The lack of physical space (e.g., not having office space) is a barrier to implementing OAAT therapy within the SC2.0 model for child and youth services. This also may pertain to rural areas	–	3	12	*We don't have offices in each region nor do we have the space to add to our current offices to accommodate how they want to implement this. (P12)*	CC1 CC4
**O1.3** Insufficient staffing	Some providers indicated that there are insufficient employees to adequately serve the amount of clients presenting for services. Others thought that SC2.0 would result in an unmanageable increase in client demands relative to staffing capabilities. This may result in challenges with the rollout	–	–	43	*Our organization is already extremely understaffed, while stepped care is supposed to help us manage waitlists and decrease wait times, we are also being told that every member of staff is to start implementing this; adding another responsibility to clinicians is going to take them from clients they're already serving. (P92)*	CC1 CC4
*Interprofessional and interorganizational misalignment*						
**O2.1** Stakeholder and management lack of support of SC2.0 model	Lack of support and disagreement between stakeholders and management about the value and evidence of SC2.0	–	–	13	*I feel that health management will not agree to the process of the Stepped 2.0 process. To understand my answer you would have to understand the power struggle between health and education that exists in the Integrated Service Delivery model. (P1123)*	–
**O2.2** Stakeholders do not have a shared understanding and vision of the SC2.0 model, which acts as a barrier to collaboration and cooperation among professionals and work settings	Each stakeholder (e.g., community partner, healthcare partners) has a unique contribution and plays an important role in the continuum of care. A unified approach stems from collaboration and a shared understanding of the model. Numerous child and youth providers felt that partners (e.g., from the school districts) were insufficiently engaged, and further education is needed to help them understand their role in the model and the role of lower intensity services	3	3	33	*All systems need to aim to be aligned with this—therefore they must talk and work together to ensure fidelity for it to work efficiently (P1208)*	CC1
**O2.3** Current organizational protocols and procedures are incongruent with the procedures of OAAT therapy sessions	Current organization procedures (e.g., information technology systems for documentation, intake assessment processes, etc.) and frameworks will create difficulties in transitioning to the provincial SC2.0 model, specifically with OAAT therapy sessions. For example, at time of interview, practices involved completing an intake and placing clients on a 7-month waitlist. Providers expressed uncertainty whether employers would support them moving away from a model that involved completion of a comprehensive intake	5	–	25	*Assessments. We currently complete an Intake Assessment for all new referrals. with SC2.0, there are no formal assessments like the one we use. Not sure if not completing our normal Intake Assessment would be supported by our employer. (P1038)*	CC1 CC4
** O2.3.1** Individuals in leadership may not have sufficient background experience	Some providers feel that "higher up" individuals had insufficient field experience to hold realistic expectations. Providers would like the organization to privilege their expertise	4	–	–	*I just hope those who are in the managerial and those director positions listen to the front-line workers because so often we're not heard and we're dealing in it. We're emersed in it every day. (P250)*	–
*Uncertainty in the organization's planning and preparations*						
**O3.1** Uncertainty in the organization's planning and preparations	Providers reported suboptimal trust in governmental organizations. Concern was expressed around the history of poorly planned provincial change initiatives, influencing providers’ perceptions of how the SC2.0 model will be executed and rolled out	6	7	15	*That being said, I don't have confidence that the government can support system wide changes that will implement stepped care properly. (P1198)*	–

Provider barriers						
Theme	Description	OAAT Interview (*N* ≥ 3)	SC Interview (*N* ≥ 3)	ISCP (*N* ≥ 10)	Representative Quote	Associated SC2.0 Core Component
*Provider resistance to the implementation of SC2.0 into practise*						
**PBar1.1** Perceived provider lack of support of SC2.0 model	Perception that a lack of support for the SC2.0 model by clinicians, stakeholders, and management will reduce the likelihood of enacting required processes or policies, and undermine implementation	–	–	16	*Not all providers agree with this method of intervention. (P1219)*	–
**PBar1.2** Difficulty overcoming complacency with current practice	Providers expressed hesitancy or resistance to OAAT therapy and SC2.0 because they are comfortable with the methods of their current practice and do not want to deviate. This may be more common among: 1) clinicians that have been practicing for an extended period of time and/or highly value traditional methods of practice (e.g., need for long-term treatment); or 2) providers who just settled into their role who may be reluctant to make additional changes	5	5	37	*Some clinical-minded professionals present with a traditional mindset of the clinical/professional is best suited to determine the needs of the client and that things cannot or should not be changed. This is a barrier I can see to implementing step care. (P1140)*	–
**PBar1.3** Difficulty accepting and adjusting to change	A belief was held that providers or colleagues struggle to adjust to change in general. This may result in hesitance or difficulty enacting the SC2.0 model. Providers felt that they will require a period of adaptation to adjust to this change process	–	–	25	*Overall, I think it will be a challenge for staff to shift their thinking and processes. (P161)*	–
**PBar1.4** Difficulty enacting change during a time when providers are already overburdened with work demands	With already high work demands (e.g., caseload), finding time to complete training and prepare/gather resources to adapt their practice to the model will be a challenge. Some providers indicated that OAAT therapy would be added to their existing caseload and create additional days of being "on call."	–	–	19	*Worry that the work of creating these additional steps will be placed on the front line workers;I worry that offering same day OAATS appointments will mean additional days of being "on call" in addition to the days we are already expected to do. (P1199)*	–
*Insufficient perceived self-efficacy to enact OAAT therapy sessions*						
**PBar2.1** Not feeling prepared and confident in delivery of skills required for OAAT therapy sessions	Some providers believe they do not know what they are doing. They may not feel confident in their skills or enacting their skills in practice	3	–	–	*When I did the online training- just the online training I found it harder to [implement OAAT into practice] because it gave me a good sense of what everything was, but I didn't really know what to do yet. (P1002)*	CC7
**PBar2.2** Providers have an insufficient awareness of accessible resources	Despite the existence of resources, providers are unaware or lack knowledge of them. A compilation of resources for provider reference would be beneficial	–	–	13	*Difficulty in knowing what all the community resources are, many groups in clinic are ending/starting/shifting (P205)*	CC2

Client barriers						
Theme	Description	OAAT Interview (*N* ≥ 3)	SC Interview (*N* ≥ 3)	ISCP (*N* ≥ 10)	Representative Quote	Associated SC2.0 core component
*Provider concern regarding universality of OAAT within SC2.0 across populations*						
**CBar1.1** Provider concern about how OAAT therapy sessions will work for individuals with complex presenting concerns	Uncertainty regarding suitability of model for clients experiencing intersectoral issues and/or complex concerns (e.g., housing issues, addiction, personality disorders)	8	–	20	*We have had a couple of moments or a couple of clients where we found out halfway through the session that this was not a good idea (P194)*	CC3 CC5
**CBar1.2** Provider concern that the SC2.0 model will not fit for child and youth clients	Difficulty understanding how the child and youth integrated service delivery model aligns with the SC2.0 model/approach, particularly given the interaction between children and youth, their families, and the school system	–	–	27	*The model does not explain how to apply with children, teenagers, their families and the schools we work with every day. It can be difficult to even know who has the autonomy of the treatment, the parent? teenager? (P1005)*	CC1 CC5
*Inequitable access to services*						
**CBar2.1** Inequity of access to services due to economic barriers	Lack of economic resources (e.g., transportation, internet, access to technology) prevents clients from being able to access the full continuum of care	4	4	14	*NB is the province with the lowest literacy rate. Having step one being a self-information style process, I can foresee many barriers from many NB'ers (P1040)*	CC1 CC6
*Incongruent expectations of service delivery*						
**CBar3.1** Client preference for or expectation of long-term therapy when attending OAAT therapy sessions	Client (or parent of client) expects or requests long-term therapy. The unmet expectation to receive long-term therapy may result in client resistance towards the SC2.0 approach	–	–	27	*Some clients expect traditional treatment and want long term support even if it is not necessary. (P1005)*	CC2 CC7 CC8
**CBar3.2** Client preference for or expectation of higher-intensity service during OAAT therapy sessions or when referred to other resources throughout the SC2.0 continuum	Client (or parent of client) expects or requests more intensive services or is told that a higher-intensity service will work better. Often, these higher-intensity services are not warranted to meet the needs of the client, but the misalignment with client expectations may lead to resistance towards the SC2.0 approach	–	–	30	*We notice that many clients expect intensive psychotherapy for situations that could be resolved by a much less intensive intervention. Also we notice that many clients ask to be seen by a psychologist, even if their situation could be answered by a practitioner from another discipline (P1011)*	CC2 CC7 CC8
*Low client capacity for change and engagement*						
**CBar4.1** Client’s may experience low readiness and motivation to engage in a service/treatment	Clients (or parents/guardians of clients) may not be ready to engage in the service. This may become apparent through low motivation and lack of willingness to participate in the service and put effort into their recovery	–	–	31	*Getting the client to see themselves as the expert of their own life when sometimes they are looking for a quick fix. (P190)*	CC6 CC9
**CBar4.2** Client difficulty accepting and adjusting to change in service to OAAT therapy sessions	Clients may have difficulty adjusting to the change because of their comfort with the approach to care that they were receiving, and/or expectations for a service. This may result in resistance towards the SC2.0 approach. Clients will need time to adjust to this change process in order to become more engaged	12	–	34	*It has taken a little while to kind of get [clients] used to the fact that things have changed. You get a few clients where they’re just so used to the old system, but that's what they just expect (P22)*	CC7 CC9
** CBar4.2.1** Client dependence on the provider	Client may become securely attached to a provider or service and may have difficulty transitioning to an OAAT therapeutic approach. Client may rely on the provider to be the expert and make decisions for them, or “fix” them	–	–	13	*Many clients seem to depend on their caseworker to guide them. A lot of clients say I don't know when trying to come up with what they need; they may feel like the professional is not helping them… some people want to be told what to do because it's easier; teens today are used to googling answers, are not asked to think of their own answers very often. (P1051)*	-

Practice benefits						
Theme	Description	OAAT Interview (*N* ≥ 3)	SC Interview (*N* ≥ 3)	ISCP (*N* ≥ 10)	Representative quote	Associated SC2.0 core component
*Increased efficiency throughout practice*						
**PBen1.1** Efficient use of time during OAAT therapy sessions	More efficient use of time with clients when implementing OAAT therapy	7	–	10	*I like the One-at-a-Time because it's not shutting the door from people coming back and getting services, but I find we waste a lot of our time chasing after clients and getting frustrated because people aren't changing (P13)*	CC7
** PBen1.1.1** Shorter assessments and treatments when using an OAAT therapeutic approach	Client assessments and treatments are shorter when using OAAT therapeutic approach in comparison to traditional non-SC2.0 service models	12	5	–	*a lot of what I do is the screening cause we have our screening is really like 26 pages when we screen new clients. They're fixing that because of OAAT. We're going to switch it to a much- from what I saw it's going to be a much cleaner. Not so much information, there's a lot of stuff that we ask that we don't probably need to. (P1013)*	–
**PBen1.2** Fewer “no shows” by using OAAT therapy throughout the SC2.0 continuum	Fewer no shows as a result of drop-in services (i.e., OAAT therapy) and offering services that are better suited to client needs. Clients rarely miss their scheduled appointments (i.e., appointments are not wasted)	–	–	16	*I think that there will be fewer wasted appointments (fewer no shows and cancellations) that arise from a client who is receiving an ill-fitting service. (P164)*	CC7
**PBen1.3** Increased general efficiency due to the SC2.0 model	Provider describes increased efficiency (i.e., generally, resources, costs to practise)	–	5	22	*More efficient and effective. (P1117)*	CC6
**PBen1.4** Clients transition in and out of care more rapidly due to timely improvements in outcomes, resulting from an OAAT therapeutic approach	Treatments occur over fewer sessions or for a shorter duration of sessions as clients receive improved outcomes sooner. In turn, more clients can be seen sooner	–	–	19	*Seeing more clients making progress in a more timely fashion. (P1018)*	
** PBen1.4.1** Provider is able to see more clients due to an OAAT therapeutic approach for service delivery	Provider can provide treatment for more clients and clients have easier access to treatment	–	–	23	*Provide some service to more clients, rather than a lot of service to a few. (P16)*	*–*
*Increased provider job satisfaction*						
**PBen2.1** Feeling of accomplishment by providing OAAT therapy sessions to clients in a time of need	Provider feels that their work is more impactful in meeting clients’ needs (e.g., making small changes with clients that results in immediate benefits). Providers feel that they are making a difference	–	–	18	*Much more satisfaction and feel like I am making a difference today (P206)*	*–*
**PBen2.2** General satisfaction	Provider describes anticipating improved satisfaction with their job, but does not elaborate	–	–	12	*Better satisfaction from the employees. (P1073)*	*–*
**PBen2.3** Greater provider morale	Increased provider morale accomplished by improved confidence, satisfaction, and work-life balance through the SC2.0 model	-	3	11	*Will result in happier staff. (P1174)*	*–*
*Reduced provider burden*						
**PBen3.1** Reduced caseloads for providers	Through the implementation of a SC2.0 model, providers anticipate having reduced caseloads, possibly related to a reduction in long-term clients on caseloads	–	3	23	*The references keep coming in people coming in for services and move them to other resources at the time, you end up with this ever-increasing caseload, higher rate of burnout people having less time to dedicate to every individual. I think it's so important to be able to manage caseloads to adequately meet the needs of people and the increasing need for mental health services. That's been present for a number of years so it's great that we finally have SC2.0 now. (P42)*	–
** PBen3.1.1** Encourages interprofessional support	SC2.0 fosters a collaborative environment among colleagues in which providers can look to each other for advice or support	–	3	–	*With clinicians, we have lunch together and stuff and sometimes it's heavy… You know, like, sometimes clients are not at a good space. It can be demanding for clinicians. So once they identify where, what support that client needs, or, like, what level they are, they can distribute it evenly, so you don't have a clinician that has a lot of cases … (P54)*	–
**PBen3.2** Sense of relief in practice due to upcoming implementation of SC2.0	Providers feel less stress, pressure, and frustration due to the change in service due to the SC2.0 approach (e.g., less pressure for clinicians/service to have all the answers or fix client concerns)	–	–	22	*Less pressure on clinicians to feel like they have to fix everything. (P1173)*	–
**PBen3.3** Client's readiness to engage in a particular service within the SC2.0 continuum reduces the burden of the provider	Provider burden is reduced because the client is ready to engage in their care and the provider does not have to "chase" or repeatedly follow up with the client	–	–	17	*People who actually want services receive them.(P1215)*	–
*Model fosters provider’s understanding of their role and engagement in the system, promoting more effective practice and tangible change*						
**PBen4.1** SC2.0 is congruent with professional standards and is felicitous for the current climate	This model encompasses the treatment style that providers believe is their perceived standard of care	–	3	–	*They are coming in, we’re helping them, they’re leaving feeling better. That’s the whole point of what we’re doing (P217)*	–
**PBen4.2** An antidote to complacency	Provider describes that the implementation of the SC2.0 model changes their past approach to practice, and jolts providers out of their old way of doing things	–	5	–	*Every now and then I will catch myself falling back into that, you know, ‘ah, what's really going on here? What’s wrong?’ and then want to dive into it. I probably still do that because I've been doing it for years, right? If can identify what's going on with you, then I can pick the best treatment for you, right? So, one of the things that this program has done for me is help me remember there's other ways to treat problems that don't involve a person coming into my office for an hour every 2 weeks. (P203)*	–
**PBen4.3** Provider readiness for change	Providers welcome the shift in practise and believes it will benefit clients and practise	–	6	–	*it's sure I think it's a transition, but I think everybody's going to get there and I think they’ll see the benefit. (P54)*	–

Client benefits						
Theme	Description	OAAT interview (*N* ≥ 3)	SC interview (*N* ≥ 3)	ISCP (*N* ≥ 10)	Representative quote	Associated SC2.0 core component
*Services are better matched to client*						
**CBen1.1** Client-centred care to meet clients’ needs and preferences	Services in the SC2.0 continuum provide clients with catered and specialized care that is targeted to their needs and preferences	–	7	41	*[referring to stepped care] it's not about the clinician and my case load and what I think they need, or don't need it really is listening to that client, that student and, and trying to figure out what do they need in this moment (P1120)*	CC9
** CBen1.1.1** Client is matched to service based on readiness and motivation	“Meets the client where they are at”; Clients are enrolled in the appropriate services that align with their level of motivation and readiness to engage in care	11	4	52	*I really liked the idea that we could meet them where they were at, at that moment, to kind of help to diffuse some of those crises rather than later on down the road (P22)*	CC9
** CBen1.1.2** Client is matched to service based on appropriate intensity for their needs and preferences	Clients are enrolled in the appropriate services, aligning with the level of intensity they need and level of commitment they are ready for. Clients have the flexibility to step up or down the continuum as needed	–	5	21	*I have this client who absolutely loves the online modules. It was like, that's all I need – I’m good to go. And yeah, I was thinking I was like, that's interesting because that's probably someone who we would have for counseling for, like, 3 to 6 sessions or more in the past. And then she took these modules on anxiety and just ran with them. Just felt like she learned so much from them. (P1014)*	CC9
**CBen1.2** Increased collaboration between client and clinician	Providers are implementing key principles into practice, particularly relating to collaboration and considering client’s needs, readiness, and preferences, rather than care being dictated by the provider or fully autonomous. Further, providers were reminded about the value of collaboration in the online courses	7	4	19	*collaboration, right, treating the client as an equal, not assuming I'm the expert and that I can tell them what to do. So I've always have that any way, that collaborative approach and and definitely trying to empower them. (P203)*	CC8
*Client-centricity*						
**CBen2.1** Client empowerment	Suggests that new services will function to empower clients to lead their own mental healthcare journeys	–	3	27	*I think they feel more empowered when they realize that, ‘Oh, you know, I have the ability to do these things by myself. (P37)*	CC5
**CBen2.2** Strengths-based approach within OAAT sessions of the Stepped Care model	Directly talks about emphasis put on the strengths of the client or that OAAT sessions takes a positive approach within the Stepped Care model	4	3	21	*If they're coming in really problem focused, trying to tease out with them when has it been less of an issue for them and what other situation in life have they been able to overcome them. How they did that, and kind of taking that to try to figure out, based on that, what do you think the next step is for with the issue that you're coming with. (P16)*	CC5
**CBen2.3** Opportune timing of treatment as clients can avail of OAAT therapy sessions within the SC2.0 model	Any response alluding to care being received at the time that the client needs it, or when they are most motivated and ready (i.e., the right care offered at the right time)	4	3	40	*They get that point of entry to the service. And if it's not you know they still made contact they've got confidence in the system, and you're able to refer to them for a higher intensity if need be (P42)*	CC6
**CBen2.4** Solution-focused OAAT therapy sessions	Addresses clients actual and practical issues. Focus is placed on the clients presenting concern, rather than “digging” for the root cause	14	5	33	*It'll be less hard for the clinician; it'll be easier because sometimes you're feeling like you're always going in circles. You're not really getting anywhere, but here it's focused. With stepped care you really focus on what’s the issue today? what do you want to work on? (P1032)*	CC7
**CBen2.5** Increased client autonomy	The client is provided with a sense of control, autonomy, and choice in their care (i.e., shared decision making). This is viewed as leading to clients who are active in their own care. A belief was held that increased autonomy would lead to better outcomes	13	3	88	*I think this model encourages people to have autonomy and with support, work to address the issues that they are having. And I think with that we have better success that way. (P217)*	CC7 CC9
** CBen2.5.1** SC2.0 prevents client dependency on the provider	Providers feel that SC2.0 encourages the client to have responsibility over their own recovery, thereby preventing dependency on the therapist for the client's well-being. Clients learn to identify their needs, promoting autonomy and independence	–	5	–	*I don't want them to be dependent on me. I don't want to take over their life, right? I'm trying to give them the skills that they need to build themselves up…(P203)*	–
*Improved overall experience with the mental health care system*						
**CBen3.1** Client satisfaction with OAAT therapy	Clients are described to enjoy OAAT therapy, leaving sessions happy and satisfied with their outcome	–	3	13	*I think clients will have a better chance of getting the care they are hoping for. (P167)*	CC8
**CBen3.2** Improved client outcomes	Clients leave treatment with better results. Discusses SC2.0 resulting in real change, improvements, and being more useful	–	3	30	*Recovery at the forefront of practice. (P128)*	CC8
**CBen3.3** Improved quality of treatment through SC2.0 model	Quality of treatment provided to clients is described to be improved from past approaches. Change to the previous system was welcomed and noted as “long overdue”	–	–	10	*A clear Framework that assists with aligning who we help, how we do it, when we do it, where we do it and why we do it. (P90)*	CC4
**CBen3.4** OAAT therapy sessions are appropriate for clients in crisis	Providers felt the model is a great approach for clients in crisis	5	–	–	*It's a really great approach for people who just are having a crisis and that's a lot of our clients (P266)*	CC7
**CBen3.5** Quicker access to care through reduced wait-lists and wait-times	Suggests that services will be more easily accessed. Clients can access services faster and therefore the wait-times are reduced, including mention of same-day services and bypassing the intake process	15	8	178	*Receive quicker service instead of waiting a significant amount of time on a waitlist. (P1098)*	CC6 CC7
** CBen3.5.1** Clients can avail of OAAT therapy and lower intensity sessions while waiting on a wait-list	The SC2.0 continuum offers clients various forms of support, even while on a waitlist for higher intensity services	–	4	–	*With the single session we’ve recently talked about what if you had a group of, you know, 5 or 6 people, and you did 4 or 5 coping skills and then if they have to sit on the waitlist, then at least they are sitting there with some skills. So, yeah, we're just sort of trying to figure out how to put it all in place. (P37)*	CC7
**CBen3.6** Increased accessibility to a greater variety of services for clients	More diverse resources and types of services will be offered through the SC2.0 continuum of care, which will offer clients informed choice when deciding on the best care option to meet their needs. Clients will feel like they have more choice in options of care that can better meet their level of readiness	–	–	64	*Knowing they have options when accessing services. (P344)*	CC6

Participants who primarily worked with adults were assigned a participant ID starting at 1 up to 999

Participants who primarily worked with children and youth were assigned participant IDs beginning at 1000

#### Insufficient resources to enact SC2.0

Providers perceived a lack of access to resources throughout the SC2.0 continuum, including peer support, guided self-help, and educational materials (Theme O1.1; *N*_SC_ = 7; *N*_ISCP_ = 104). Availability of these resources were also noted to be reliant on the region, where the options made available to those in one region, such as an urban location, may not be available to individuals in another region, such as a rural location, and vice versa (Theme O1.1.1; *N*_SC_ = 3):

*“Part of being able to deliver Stepped Care is knowing what Stepped Care is in the sense of what is available to people… You can't show us in a Stepped Care training exactly what's going on in our region, because it's unique to our region.” (P217)*

The organization’s ability to enact SC2.0 may also be impacted by insufficient staffing (Theme O1.3; *N*_ISCP_ = 43) and a lack of spaces for child and youth providers to conduct OAAT therapy sessions impedes rapid access to drop-in services (Theme O1.2; *N*_SC_ = 3; *N*_ISCP_ = 12).

#### Interprofessional and interorganizational misalignment

Some providers indicated that lack of support and disagreement with the model from management and key stakeholders may impede implementation (Theme O2.1; *N*_ISCP_ = 13). Importantly, providers mentioned a mixed understanding amongst stakeholders of what a provincial SC2.0 model could look like that could negatively impact the collaborative process across the system. As a result, providers recommended further education for all stakeholders (e.g., A&MH services and school districts; Theme O2.2; *N*_SC_ = 3; *N*_OAAT_ = 3; *N*_ISCP_ = 33):

*“All systems need to aim to be aligned with this - therefore they must talk and work together to ensure fidelity for it to work efficiently.” (P1208)*

Providers noted that current organizational protocols and documentation processes did not always align well with the implementation of OAAT therapy in practice (Theme O2.3; *N*_OAAT_ = 5; *N*_ISCP_ = 25). This could include charting requirements in information technology systems, and mandated intake assessment processes.

#### Uncertainty in organization planning

Providers expressed uncertainty towards the organization’s ability to successfully plan and prepare for the implementation of SC2.0 due to past provincial change initiatives which had been less than optimally planned or executed (Theme O3.1; *N*_SC_ = 7; *N*_OAAT_ = 6; *N*_ISCP_ = 15).

*“That being said, I don't have confidence that the government can support system wide changes that will implement stepped care properly.” (P1198)*

### Provider barriers to implementing SC2.0 in practice

Two themes emerged from open-ended responses (*N*_ISCP_ = 141) and interviews that pertained to provider-related barriers: (1) resistance to the implementation of SC2.0 into practice; and (2) perceived lack of self-efficacy to enact OAAT therapy sessions.

#### Resistance to the implementation of SC2.0 into practice

Some providers reported that a lack of support for the SC2.0 model would directly cause strain on the implementation process (Theme PBar1.1; *N*_ISCP_ = 16). Further, there may be providers who are complacent in their practice, resulting in resistance to this change (Theme PBar1.2; *N*_SC_ = 5; *N*_OAAT_ = 5; *N*_ISCP_ = 37):

*“As it is a newer philosophy, it will be important for providers to change their perspective, and adopt the single-session mindset. This may pose a challenge to clinicians who have been operating a certain way for a long time.” (P42)*

#### Perceived lack of self-efficacy to enact OAAT

Some providers indicated a lack of confidence in their skills to enact OAAT therapy with clients, which was compounded by insufficient training in formal counselling (Theme PBar2.1; *N*_OAAT_ = 3). Other providers noted a lack of confidence in their knowledge of available resources (Theme PBar2.2; *N*_ISCP_ = 13), and wished to see a compilation of accessible resources for reference.

*“When I did the online training- just the online training I found it harder to [implement OAAT into practice] because it gave me a good sense of what everything was, but I didn't really know what to do yet.” (P1002)*

### Client barriers to implementing SC2.0 in practice

Four client-related themes emerged from open-ended responses (*N*_ISCP_ = 156) and qualitative interviews: (1) concern regarding the universality of SC2.0 across populations; (2) inequitable access to services; (3) incongruent client expectations of service delivery; and (4) limited client capacity for change and engagement in care.

#### Concern regarding the universality of OAAT within SC2.0.

Providers felt uncertain about how to implement with diverse clientele and questioned the universality of OAAT within the SC2.0 model. For example, child and youth providers were concerned that OAAT sessions would not suit the needs of their clients (Theme CBar1.2; *N*_ISCP_ = 27). Moreover, providers were concerned that complex cases (e.g., housing issues, addiction, and personality disorders) would “slip through the cracks” with the use of OAAT therapy (Theme CBar1.1; *N*_OAAT_ = 8; *N*_ISCP_ = 20):

*“People with addictions want to change but when they are discharged some return to their old patterns of living. I work in addictions, and some clients may have a hard time with any sort of care at the beginning stages. Could be started when the client is ready.” (P216)*

#### Inequitable access to services

Providers felt that some clients may be unable to access services within the SC2.0 continuum due to socioeconomic challenges, including limited access to technology (e.g., internet, computers, telephones), transportation, and other resources required for treatment (Theme CBar2.1; *N*_SC_ = 4; *N*_OAAT_ = 4; *N*_ISCP_ = 14).

*“Technology is a big one- there are a huge amount of barriers recommending people to download an app, or input data. In rural settings people do not have consistent internet and tech literacy can be low.” (P1133)*

#### Incongruent client expectations of service delivery

Providers noted that some clients have a preference or expectation for long-term therapy (Theme CBar3.1; *N*_ISCP_ = 27) or higher-intensity services (Theme CBar3.2; *N*_ISCP_ = 30), and that this expectation is potentially be incongruent with an SC2.0 approach to treatment:

*“Clients expect that when they come to the centre with issues, they want the most intensive treatment first (e.g. individual therapy), and don't consider the other stepped care options.” (P1002)*

#### Limited client capacity for change and engagement in care

Providers reported that clients may have low readiness and motivation to engage in treatment, and dedicate insufficient effort into their recovery (Theme CBar4.1; *N*_ISCP_ = 31). Similarly, a client’s difficulty accepting and adjusting to the change in provincial services (Theme CBar4.2; *N*_OAAT_ = 12; *N*_ISCP_ = 34) was viewed as a barrier to participation in the model:

*“You get a few clients where they’re just so used to the old system, but that's what they just expect…. so sometimes kind of changing and challenging that and those expectations, I mean, sometimes challenging, and other times it goes quite well.” (P22)*

### Facilitators to implementing SC2.0 in practice

**Fig. 4:**
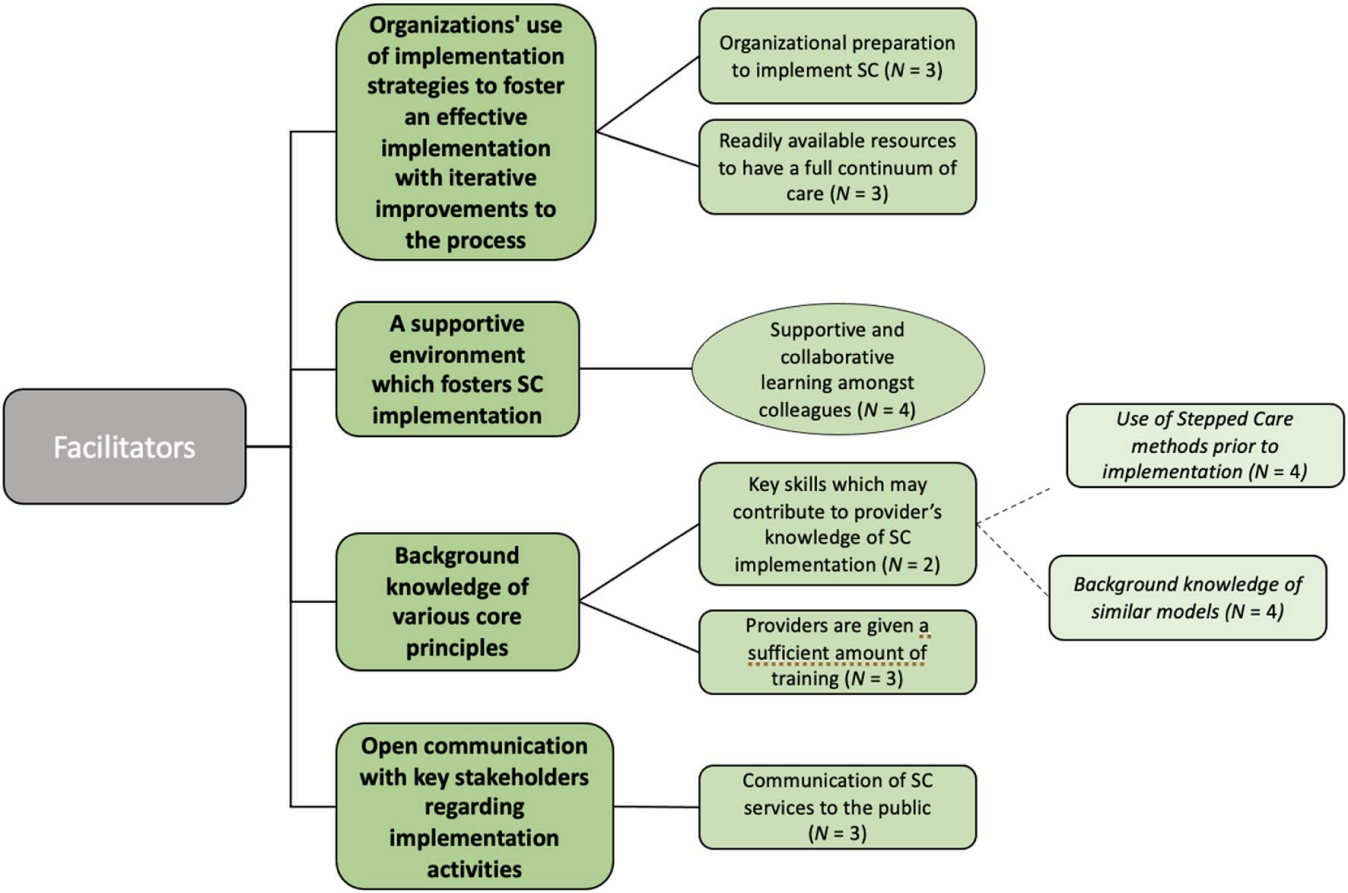
Summary of results for qualitative interviews: perceived facilitators. Provider responses were thematically analyzed into themes (bold), codes, and subcodes (*italics*). Codes which were observed in both qualitative interviews and open-ended responses are encased in circles. Codes which were observed only in qualitative interviews are encased in boxes

Four facilitators of implementation were identified through qualitative interviews, refer to Fig. 4. Facilitators included: (1) Organizational preparation (e.g., creating a new clinical lead role for OAAT therapy and hiring a change management specialist) and use of evidence-based implementation strategies (e.g., identifying champions, planning and preparing for rollout, and using data for continuous improvement) were found to be helpful (*N*_SC_ = 3); (2) Cultivation of a supportive environment which fostered the implementation of SC2.0 (*N*_SC_ = 4). One provider explained:

*“I think everybody is moving in a common direction. We all have the same goal so this might be a sort of roadmap that we need in order to stay on track towards that goal together because otherwise everybody's lost.” (P42)*

(3) Gaining familiarity with the core values and concepts (e.g., recovery-oriented practice and client-centered care; *N*_SC_ = 4) of SC2.0, and complimentary models of care (e.g., solution-focused principles) (*N*_SC_ = 6); and (4) open communication with key stakeholders. Specifically, providers implied that clear and transparent communication of SC2.0 services (e.g., drop-in services) helped to communicate the change in service delivery to the public (*N*_SC_ = 3).

### Perceived benefits to clinical practice associated with implementing SC2.0

**Fig. 5:**
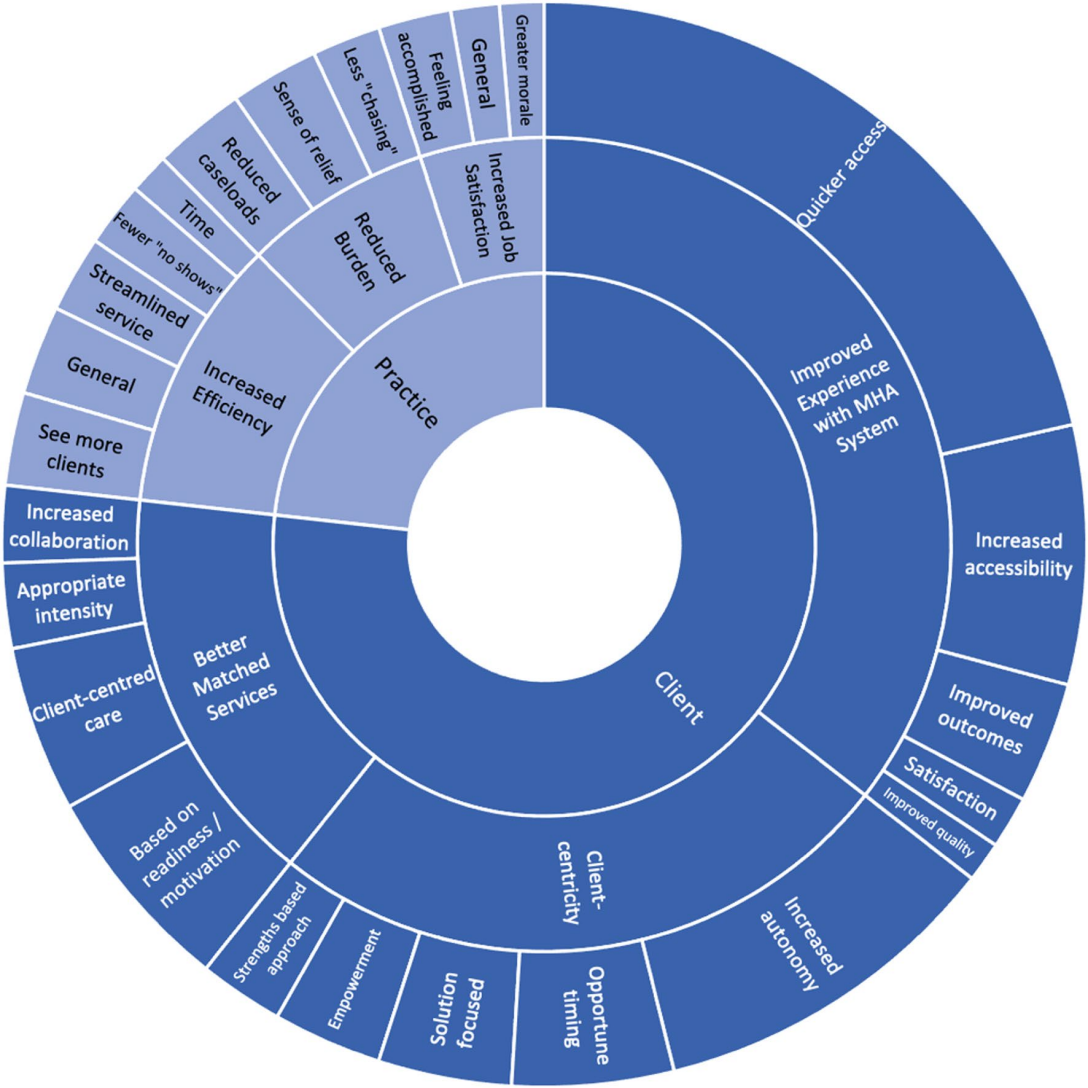
Summary of results for open-ended responses: perceived benefits. Innermost section represents the respective stakeholder the barrier would affect. The middle section represents the overarching theme, followed by the code in the outermost section. Size of each box is determined by the number of provider responses per code

Extracted benefits from open-ended responses and qualitative interviews can be located in Fig. 5. Four themes emerged across open-ended responses (*N*_ISCP_ = 300) and qualitative interviews: (1) increased efficiency throughout practice; (2) increased job satisfaction; (3) reduced provider burden; and (4) model fosters role coherence, and promotes effective practice and tangible change.

#### Increased efficiency through practice

Increased practice efficiency (Theme PBen1.3; *N*_SC_ = 5; *N*_ISCP_ = 22) was anticipated to result in improved outcomes for clients, providers, and the system. Providers anticipated an efficient use of time when implementing OAAT therapy (Theme PBen1.1; *N*_OAAT_ = 7; *N*_ISCP_ = 10), including shorter screening processes and brief courses of treatment (Theme PBen1.1.1; *N*_OAAT_ = 12; *N*_SC_ = 5), which would allow for a more streamlined delivery of care (Theme PBen1.4; *N*_ISCP_ = 19):

*“[referring to past methods] It’s really like 26 pages when we screen new clients…There's a lot of stuff that we ask that we don't probably need to.” (P1074)*

#### Increased job satisfaction

Providers reported that the implementation of SC2.0 may enhance job satisfaction. Providers anticipate feelings of accomplishment (Theme PBen2.1; *N*_ISCP_ = 18) by delivering services that they felt better met client needs. Providers also expressed a potential for improved job satisfaction (Theme PBen2.2; *N*_ISCP_ = 12) and greater workplace morale (Theme PBen2.3; *N*_SC_ = 3; *N*_ISCP_ = 11).

#### Reduced provider burden

Providers felt that they would spend less time “chasing” resistant clients, and that “no show” behaviour would be decreased by empowering clients and involving them in decisions about their care (Theme PBen1.2; *N*_ISCP_ = 16). Specific to OAAT therapy within the SC2.0 model, one provider noted:

*“It's not shutting the door from people coming back and getting services, but I find a lot of- we waste a lot of our time chasing after clients and getting frustrated because people aren't changing.” (P13)*

#### Model promotes effective practice, coherence in professional role, and tangible change

Providers believed that implementing SC2.0 would result in fewer long-term clients and inappropriate referrals which would reduce burden and caseloads (Theme PBen3.1; *N*_SC_ = 3; *N*_ISCP_ = 23). Further, providers indicated that SC2.0 fosters a collaborative environment among colleagues that facilitated the provision of advice and support (Theme PBen3.1.1; *N*_SC_ = 3). This could range from support with complex cases, to referrals within the community that would better meet client needs:

*“It can be demanding for clinicians. So once they identify where, what support that client needs, or, like, what level they are, they can distribute it evenly, so you don't have a clinician that has a lot of cases.” (P54)*

Providers believed that implementing SC2.0 in their practice would result in less stress, pressure, and frustration delivering services (Theme PBen3.2; *N*_ISCP_ = 22), due to greater client engagement and reduced burden of clients who did not attend scheduled appointments (Theme PBen3.3; *N*_ISCP_ = 17).

Providers welcomed the shift towards an SC2.0 approach, perceived it to be well suited for the current mental healthcare climate (Theme PBen4.1; *N*_SC_ = 3), and believed that it would benefit clients and reinvigorate their practice (Theme PBen4.3; *N*_SC_ = 6):

“They are coming in, we’re helping them, they’re leaving feeling better. That’s the whole point of what we’re doing.” (P217)

SC2.0 was described as an antidote to complacency (Theme PBen4.2; *N*_SC_ = 5), and the adoption of an SC2.0 model changed providers’ past approach to practice by promoting new ways of thinking:

“One of the things that this program has done for me is help me remember there’s other ways to treat problems that don’t involve a person coming into my office for an hour every 2 weeks.” (P203)

### Perceived client benefits associated with implementing stepped care 2.0

Open-ended responses (*N*_ISCP_ = 300) and qualitative interviews resulted in three themes: (1) better matched services; (2) benefits of client-centricity; and (3) improved client experiences with the NB A&MH system.

#### Better matched services

Providers reported that SC2.0 promotes client-centred care to meet a client’s needs and preferences (Theme CBen1.1; *N*_SC_ = 7; *N*_ISCP_ = 41), and noted increased collaboration between providers and clients when using the SC2.0 model (Theme CBen1.2; *N*_SC_ = 4; *N*_OAAT_ = 7; *N*_ISCP_ = 19). Shared care decision-making was believed to result in the selection of the most appropriate services that aligned with the level of intensity clients need and level of commitment they are ready for (Theme CBen1.1.2; *N*_SC_ = 5; *N*_ISCP_ = 21). Providers also noted the need to consider and respect client readiness to engage in a service (Theme CBen1.1.1; *N*_SC_ = 4; *N*_OAAT_ = 11; *N*_ISCP_ = 52) and meet clients “where they are at”:

“It really is listening to that client, that student and, and trying to figure out what do they need in this moment.” (P1120)

#### Benefits of client-centricity

Providers identified solution-focused sessions (Theme CBen2.4; *N*_SC_ = 5; *N*_OAAT_ = 14; *N*_ISCP_ = 33), and a strengths-based approach (Theme CBen2.2; *N*_SC_ = 3; *N*_OAAT_ = 4; *N*_ISCP_ = 21) as helpful in identifying and building on the client’s existing skills:

“Meet the client where they're at, keep it client centered, remind them of their strengths.” (P203)

Ultimately, client-centric approaches endorse empowerment (Theme CBen2.1; *N*_SC_ = 3; *N*_ISCP_ = 27), as clients lead their own mental health journeys. Providers also noted that OAAT therapy delivered within the context of SC2.0 promoted access to care at the opportune time by allowing clients open access to services which they may avail of for as many sessions they wish during their time of need (Theme CBen2.3; *N*_SC_ = 3; *N*_OAAT_ = 4; *N*_ISCP_ = 40).

#### Improved client experiences with the NB A&MH system

Providers perceived that the adoption of an SC2.0 model would facilitate timely access to services (Theme CBen3.5; *N*_SC_ = 8; *N*_OAAT_ = 15; *N*_ISCP_ = 178), and increase availability of diverse services (e.g., lower intensity programming, peer support; Theme CBen3.6; *N*_ISCP_ = 64), including while waiting for higher intensity services (Theme CBen3.5.1; *N*_SC_ = 4). SC2.0 services were perceived to improve treatment quality compared to past models (Theme CBen3.3; *N*_ISCP_ = 10), and result in better outcomes for clients (Theme CBen3.2; *N*_SC_ = 3; *N*_ISCP_ = 30):

“Increasing success in interventions and in client improvement.” (P1143)

## Discussion

Providers who worked within A&MH services in NB, Canada received training in SC2.0 and One-at-a-Time therapy in preparation for a system change initiative. Our team conducted a mixed-methods observational implementation study to gain a better understanding of: (1) acceptability and feasibility of a provincial stepped care framework for A&MH services; and (2) perceived barriers, facilitators and benefits from enacting a provincial stepped care model. Surveys were completed by providers to gather demographic information, and quantify provider views of acceptability, feasibility, readiness, motivation, and organizational commitment to implement a provincial SC2.0 framework. Interviews and open-ended questions served as an adjunct to quantitative surveys and were used to elaborate on the perceived barriers and benefits associated with integrating SC2.0 into practice.

### Acceptability of stepped care in the addictions and mental health system

The multi-construct Theoretical Framework of Acceptability [37] provided a lens to understand acceptability. Providers, and particularly adult providers, endorsed a high level of acceptability towards implementing a provincial stepped care model, as evidenced by: (1) welcoming the model and endorsing its value; (2) identifying personal (e.g., maintaining relationships) and professional (e.g., efficiency) benefits of adoption; (3) indicating ease of implementation without associated opportunity costs; and (4) reporting motivation and intention to incorporate a stepped care approach within practice. Notably, providers who served child and youth populations reported lower levels of acceptability, appropriateness, and feasibility of implementing SC2.0 relative to providers who served adult populations, likely stemming from barriers related to: (1) perceived insufficient space to deliver OAAT therapy sessions; (2) concerns that OAAT therapy within the context of SC2.0 may not best meet the needs of child and youth clients; and (3) challenges in the shared vision and understanding of SC2.0 with partnering organizations. It is not surprising that acceptability, appropriateness, and feasibility of implementation was higher among providers who served adult populations given that these providers work in community A&MH clinics, and do not work as a part of an integrated service delivery model with diverse partners who require a thorough understanding of OAAT therapy and SC2.0.

Overall, observed level of acceptability for the implementation of SC2.0 into the A&MH system is encouraging given that intervention acceptability from the perspectives of healthcare professionals has been proposed to impact implementation, uptake, adherence, intended outcomes, and overall effectiveness [43–46]. That said, it is important to note that responses pertained to acceptability of the overall SC2.0 system and not its core components, each of which may require a substantial undertaking to implement and may vary from overall acceptability of the SC2.0 model.

### Perceived benefits to adopting stepped care

Providers who worked within A&MH services anticipated that the implementation of SC2.0 would increase accessibility to resources at varying levels of intensity. Expanding access to mental health services within practice has been observed to improve the morale and satisfaction of providers who work within primary care [47]. Consistent with this, providers reported making a meaningful impact by addressing client needs using OAAT therapy sessions in the present study. Open access to a continuum of care options for A&MH services has also been shown to effectively reduce wait times for treatment and improve interprofessional communication [3, 48].

Adoption of SC2.0 in practice was anticipated to facilitate expedient access to A&MH care. Early intervention in mental healthcare has been shown to improve client outcomes and reduce the likelihood of accessing emergent care [49, 50]. SC2.0 provides a framework for organizing a continuum of integrated services and resources to meet the needs and preferences of clients who experience A&MH concerns, allowing for the provision of the right care at the right time. Using an OAAT approach, the client’s top of mind concern is the focus of the intervention and action plans are based on their existing strengths, preference, and level of readiness to engage with an intervention. Recent research has indicated that privileging client preferences in treatment increased motivation to participate in therapeutic interventions and reduced drop-out rates [51]. Of interest, providers anticipated that implementing SC2.0 in practice would lead to similar benefits for clients who they serve.

### Overcoming perceived barriers to adopting a provincial stepped care model

#### Adequately resourcing a stepped care continuum in A&MH services

Consistent with the second core component of SC2.0 [2], providers recognized the importance of adequately resourcing a stepped care continuum of services, and reported insufficient resources within the current system to adequately populate a continuum of care for clients. Relatedly, providers noted insufficient staffing within the organizations to provide adequate services to clients. In accordance with the capability, opportunity, motivation model of behaviour (COM-B) [52] insufficient resources to populate a continuum of care could represent barriers of psychological capability (e.g., insufficient awareness of formal and informal resources that exist within the system), and physical opportunity (e.g., insufficient materials, such as service maps). In our team’s experience, most organizations or jurisdictions that have implemented the SC2.0 model have conducted a comprehensive resource mapping exercise and developed a representation of these services and resources that can be used by providers and people accessing services. This process is a strategy to overcome barriers of psychological capabilities and physical opportunities. Of interest, the resource mapping exercise tends to illuminate greater breadth and depth of resources than would have been anticipated. Awareness and uptake of resources such as service maps could be implemented in accordance with Expert Recommendations for Implementing Change (ERIC), including: (1) training and educating stakeholders; and (2) adapting and tailoring service maps to regional contexts [53, 54]. Effective implementation of such service maps may also require change to clinical infrastructure (e.g., change to charting and referral systems).

#### Adjusting expectations during a provincial practice change

Barriers noted by providers highlighted the importance of setting and managing appropriate expectations. A portion of providers noted resistance towards enacting a change in practice due to an expectation that the change would not be sustained in the organization (i.e., previous unsuccessful provincial initiatives left some providers hesitant to commit to SC2.0). This would represent a barrier in reflective motivation [52] perpetuated by beliefs about the Consolidated Framework for Implementation Research (CFIR) domain of organization culture for sustaining change initiatives [55]. Other providers noted comfort in their current method of care delivery, and a reluctance to accept and adjust to a change in their practice. This is also reflective of suboptimal reflective motivation. Several strategies may be beneficial for overcoming such barriers to improve reflective motivation, including: (1) preparing champions to strengthen perceived organizational commitment; (2) reframing beliefs through the promotion of SC2.0 values (e.g., pros and cons) given that SC2.0 aligned with provider professional standards of care, and was deemed acceptable, applicable, and feasible to implement in practice [56]; and (3) develop and communicate a formal implementation blueprint that includes a formal plan for sustainability [57].

Providers noted that client expectations for services may represent one barrier to implementing SC2.0 in practice. Specifically, providers indicated that many clients expect high-intensity services for extended periods of time which may interfere with effective matching of services across a continuum of care. This expectation is indicative of a barrier of social opportunity (i.e., a perceived societal norm, long term or ongoing psychotherapy is the gold standard, that may interfere with offering client lower-intensity care options) [52]. It may be pertinent to adjust such social norms through the provision of knowledge and social comparisons delivered by a credible source [56]. For example, clients with more complex challenges do not always require intense services, as indicated by: (1) results from a meta-analysis indicated that individuals with severe depression experienced equivalent clinical improvement with low and high intensity offerings [58]; (2) therapeutic outcomes did not vary among clients with depressive and anxiety disorders randomized to receive low or high intensity cognitive-behavioural therapies [59], see also [60]; (3) low-intensity interventions are effective and acceptable for older adults and may have greater treatment engagement [61]; and (4) many sites that implement stepped care have diverse care pathways that lead to the delivery of relatively few high intensity services [62]. It may also be helpful to emphasize that in SC2.0, people’s level of readiness and preferences guides service delivery and planning which is consistent with core component 8 that care is flexible, collaborative and guided by data [2].

Of interest, providers questioned the universality of OAAT within the SC2.0 model and noted concern about complex clients slipping through the cracks. It is encouraging that providers were mindful of this important consideration. The implementation of continuous progress monitoring may be effective in addressing this barrier given that it has proven beneficial to identify clients who are not progressing as expected and inform timely changes in the care plan [63]. Providers also questioned equitability of access to care within the SC2.0 model. This highlights the importance of incorporating health equity domains that target culturally-relevant factors of recipients, patient-provider interactions, and societal context into the formal implementation of SC2.0 [64].

#### Organizational and stakeholder incongruence with OAAT therapy and SC2.0

Providers noted two important areas of incongruence with SC2.0. First, providers felt that organizational protocols (e.g., documentation and intake assessments) did not align with OAAT therapy, which could make delivery of Core Component 7 within the SC2.0 model difficult, refer to Fig. 2. Further, providers felt that system stakeholders, including partnering community services (e.g., corrections, school districts), did not adequately understand the value of OAAT therapy and their role in a provincial SC2.0 approach. Since data collection concluded, the core project team in NB guided a review of operational guidelines to help correct inconsistencies between organizational protocols and an OAAT therapy approach. This review resulted in modifications to the clinical documentation system to reflect OAAT therapy sessions. Further, OAAT therapy sessions were made available through self-referral without the requirement to complete an intake assessment. With respect to stakeholders’ understanding of the model and their role within the continuum of care, the core project team is developing a plan to: (1) educate community partners on the SC2.0 model and their role in the continuum of care; and (2) consult community partners on potential barriers and challenges to address in rolling out a united approach to the provincial SC2.0 model. These actions align well with co-designing the SC2.0 system with diverse perspectives and experiences (Core Component 1) [2], and ERIC strategies, including conducting educational outreach visits and needs assessments [53].

## Strengths

There are several strengths to the present study. First, there was a diverse sample that was representative of providers who served child and youth, and adult populations, from a variety of locations, such as healthcare authorities, school districts and urban and rural settings. Second, the present study used a mixed methods approach that triangulated quantitative and qualitative data that led to a more nuanced understanding of the anticipated benefits, barriers and facilitators to implementing SC2.0. Third, recognized determinant frameworks within implementation science (e.g., COM-B; ERIC; Theoretical Framework of Acceptability) were drawn from to help contextualize the observed results and inform implementation efforts.

## Limitations

Several limitations should be considered when interpreting results observed in this study. First, results pertained to the anticipated acceptability, benefits, barriers and facilitators of the implementation of SC2.0 within A&MH services (i.e., results were collected before full implementation) and may not reflect retrospective acceptability, benefits, barriers and facilitators. Second, provider tendency to respond in a socially desirable manner was not evaluated, raising the potential for response bias. This was mitigated by reducing the potential for coercion (e.g., data were collected by a research team external to the provincial implementation team, was anonymized, and only aggregate results were reported to stakeholders). Third, responses were obtained with regard to the SC2.0 model in its entirety rather than each core component. As such, it is unclear which benefits, barriers, and facilitators would be most important when implementing a given core component(s). Fourth, the present study did not include healthcare professionals who play an important role in the planning and delivery of addiction and mental health services across the continuum of care. For example, psychiatrists, paediatricians, primary care physicians, and psychologists working within independent practice were not included. Of note, the government of NB engaged in regular consultation with many of these groups throughout the implementation process, and are currently preparing for the rollout of SC2.0 for community partners and other interested parties (e.g., indigenous groups). Fifth, data were collected from approximately half of the work force within A&MH in NB, and we obtained a response rate of 54% for quantitative surveys. Therefore, we cannot rule out the potential for selection bias. Finally, qualitative data did not contain sub-population analysis; however, prominent codes which applied to a sub-population were specified in resulting themes.

## Future directions

Findings of this study will inform the identification of tailored implementation strategies that are fit for purpose to address barriers and emphasize enablers to using SC2.0 in practice [53]. Results of this study are also being fed back to NB to improve the ongoing implementation with the goal of improving patient experiences within A&MH services. Now that OAAT therapy has been successfully implemented in community services across the province and sustainability measures (e.g., service monitoring and a community of practice) are in place, the focus has turned to the provincial adoption and implementation of a SC2.0 continuum of services. It is recognised that a provincial implementation of a system wide SC2.0 continuum will be a multi-phase project, spanning 2–3 years. The work for the first 12 to 18 months involved the creation of a provincial steering committee that guides the co-design of the provincial SC2.0 continuum. This committee includes a broad spectrum of representation from government departments, health authorities, community agencies, indigenous communities, and individuals with lived and living experience in A&MH. The Steering Committee informs and guides working groups that focus on: (1) creating shared guiding principles; (2) developing a communication and engagement strategy; (3) co-designing and populating the SC2.0 continuum for NB; (4) updating operational guidelines to reflect SC2.0; (5) creating integrated care pathways; (6) developing an evaluation framework; and (7) creating a SC2.0 community of practice. Ongoing consultation and collaboration with Stepped Care Solutions, Memorial University, other jurisdictions implementing Stepped Care models and the Mental Health Commission of Canada will continue throughout this process.

The process of engagement and communication about Stepped Care implementation, as well as the consultation and collaboration in the co-design of the model is now underway. While this work is critical to the adoption of a SC2.0 continuum, its focus is at a systems level and will not immediately improve services or address some of the identified gaps. Several smaller scale direct service improvement projects are underway simultaneously to the implementation of SC2.0, including (1) implementation of an Outpatient Withdrawal Management Service so that individuals who meet the criteria do not need to go to a bed-based facility; (2) creation of a provincial substance use and mental health helpline that focuses on providing information (3) enhancing system navigation; (4) support and crisis management when needed; and (5) the ongoing expansion and improvement of online information, services, and support located on the provincial Bridge the Gapp website. Provincial implementation of projects such as these typically require 1–2 years from start of planning to successful implementation.

The implementation of OAAT therapy within A&MH services represents a common trade-off between enhancing rapid access to care with less robust screening, and ensuring client safety through comprehensive risk assessment. Facilitating rapid access in this manner requires a shift in the risk paradigm, such that it is distributed across the system, refer to core component #3 in Fig. 2. Another way to overcome this trade-off is through the initiation of measurement-based care given that continuous progress monitoring using standardized scales has consistently been observed to improve trajectories of care, particularly among those who are at risk of deterioration [63, 65]. As such, initiating measurement-based care represents one key aspect of SC2.0 implementation in NB that will help ensure that clients monitor their progress over time, and receive thoughtful deviations in care as appropriate. Resources and personnel have been committed to enact this core component within the provinces approach to implementing Stepped Care 2.0. A system evaluation framework will also be needed to ensure the Stepped Care continuum and its pathways are directing clients to the right service and that they have access to that service. While Stepped Care implementation is in the early stages, these issues are top of mind in implementation planning. The electronic medical record system for the provincial A&MH Services in NB is due for a significant update and plans are being developed for how best to embed these elements within the electronic record and a client’s individual profile.

## Conclusions

The present study sought to identify the perceived benefits, barriers, and facilitators of implementing SC2.0 in a provincial practice change. The SC2.0 model was congruent with providers’ standards of care and perceived as advantageous to the organization, providers, and clients. Of note, the implementation of SC2.0 may only be feasible if sufficient resources are available to populate the continuum of care. Results observed in this study may help inform other organizations looking to enact large-scale change, and promote effective and sustainable care through the implementation of stepped care for A&MH services.

## Data Availability

De-identified data will be made available to researchers who provide a methodologically sound proposal for the purpose of achieving the aims of the approved proposal. Data sharing will be enacted with a data-transfer agreement between the sending and receiving institutions. Proposals should be directed to the corresponding author.

## Abbreviations

AAFI: Acceptability, appropriateness, and feasibility of intervention
A&MH: Addiction and Mental Health
COC-18: Commitment to organizational change-18
ERIC: Expert Recommendation for Implementing Change
NB: New Brunswick
OAAT therapy: One-at-a-Time therapy
ROC-25: Readiness for organizational change-25
SC2.0: Stepped Care 2.0

## References

[1] O’Donohue WT, Draper C, Draper C, O'Donohue WT (2011). The case for evidence-based stepped care as part of a reformed delivery system. Stepped care and e-health: practical applications to behavioral disorders.

[2] Cornish P (2020). Stepped care 2.0: a paradigm shift in mental health.

[3] Mental Health Commission of Canada (2019). Newfoundland and labrador stepped care 2.0 e-mental health demonstration project.

[4] Carey S, Jaouich A, Churchill A, Cornish P, Impey D, Kim M. Stepped Care 2.0 revised implementation guide. Ottawa, ON: Mental Health Commission of Canada; 2021.

[5] Zhu M, Hong RH, Yang T, Yang X, Wang X, Liu J (2021). The efficacy of measurement-based care for depressive disorders: systematic review and meta-analysis of randomized controlled trials. J Clin Psychiatry..

[6] Parikh A, Fristad MA, Axelson D, Krishna R (2020). Evidence base for measurement-based care in child and adolescent psychiatry. Child Adolesc Psychiatr Clin N Am.

[7] DeSimone J, Hansen BR (2023). The impact of measurement-based care in psychiatry: an integrative review. J Am Psychiatr Nurses Assoc.

[8] Bertuzzi V, Fratini G, Tarquinio C, Cannistra F, Granese V, Giusti EM (2021). Single-session therapy by appointment for the treatment of anxiety disorders in youth and adults: a systematic review of the literature. Front Psychol.

[9] Hoyt MF, Bobele M, Slive A, Young J, Talmon M (2018). Walk-in and by-appointment single-sessions now and in the future. Single-session therapy by walk-in or appointment: administrative, clinical, and supervisory aspects of one-at-a-time services.

[10] Schleider JL, Weisz JR (2017). Little treatments, promising effects? Meta-analysis of single-session interventions for youth psychiatric problems. J Am Acad Child Adolesc Psychiatry.

[11] Rodda SN, Lubman DI, Jackson AC, Dowling NA (2017). Improved outcomes following a single session web-based intervention for problem gambling. J Gambl Stud.

[12] Mental Health Commission of Canada (2015). Guidelines for recovery-oriented practice.

[13] National Institute for Health and Clinical Excellence. Obsessive-compulsive disorder: Core interventions in the treatment of obsessive-compulsive disorder and body dysmorphic disorder: National Institute for Health and Clinical Excellence. 2005. https://www.nice.org.uk/guidance/cg31. Accessed 16 July 2023. 31886982

[14] National institute for health and clinical excellence. Depression in adults: recognition and management: National Institute for Health and Clinical Excellence. 2009. https://www.nice.org.uk/guidance/cg90. Accessed 16 July 2023. 31990491

[15] National Institute for Health and Clinical Excellence. Common mental health problems: identification and pathways to care: National Institute for Health and Clinical Excellence. 2011. https://www.nice.org.uk/guidance/cg123. Accessed 16 July 2023.

[16] Boyd L, Baker E, Reilly J (2019). Impact of a progressive stepped care approach in an improving access to psychological therapies service: An observational study. PLoS ONE..

[17] Collins P, Walsh Z, Walsh A, Corbett A, Finnegan R, Murphy S (2020). A 360° evaluation of stepped-care psychotherapy: APSI yrs 4–5. Ment Health Rev J.

[18] Firth N, Barkham M, Kellett S (2015). The clinical effectiveness of stepped care systems for depression in working age adults: a systematic review. J Affect Disord.

[19] Gyani A, Shafran R, Layard R, Clark DM (2013). Enhancing recovery rates: lessons from year one of IAPT. Behav Res Ther.

[20] Ho FY-Y, Yeung W-F, Ng TH-Y, Chan CS (2016). The efficacy and cost-effectiveness of stepped care prevention and treatment for depressive and/or anxiety disorders: a systematic review and meta-analysis. Sci Rep..

[21] van Straten A, Hill J, Richards DA, Cuijpers P (2015). Stepped care treatment delivery for depression: a systematic review and meta-analysis. Psychol Med.

[22] Morse AK, Sercombe J, Askovic M, Fisher A, Marel C, Chatterton ML (2023). Systematic review of the efficacy, effectiveness, and cost-effectiveness of stepped-care interventions for the prevention and treatment of problematic substance use. J Subst Abuse Treat.

[23] Berger M, Fernando S, Churchill A, Cornish P, Henderson J, Shah J (2022). Scoping review of stepped care interventions for mental health and substance use service delivery to youth and young adults. Early Interv Psychiatry.

[24] Mental Health Commission of Canada, Stepped Care Solutions, Government of Northwest Territories (2023). Partnering together for person-and family-centric care: the Northwest territories stepped care 2.0 final report.

[25] Hoyt MF, Bobele M, Slive A, Young J, Talmon M (2018). Single-session/one-at-a-time walk-in therapy. Single-session therapy by walk-in or appointment: administrative, clinical, and supervisory aspects of one-at-a-time services.

[26] Hymmen P, Stalker CA, Cait CA (2013). The case for single-session therapy: does the empirical evidence support the increased prevalence of this service delivery model?. J Ment Health.

[27] Stalker CA, Riemer M, Cait CA, Horton S, Booton J, Josling L (2016). A comparison of walk-in counselling and the wait list model for delivering counselling services. J Ment Health.

[28] Riemer M, Stalker CA, Dittmer L, Cait C-A, Horton S, Kermani N (2018). The walk-in counselling model of service delivery: who benefits most?. Can J Commun Ment Health.

[29] Ewen V, Mushquash AR, Mushquash CJ, Bailey SK, Haggarty JM, Stones MJ (2018). Single-session therapy in outpatient mental health services: examining the effect on mental health symptoms and functioning. Soc Work Ment Health.

[30] Statistics Canada (2022). 2021 census of population.

[31] Government of New Brunswick (2021). Inter-departmental addiction and mental health action plan: priority areas for 2021–2025.

[32] Government of New Brunswick (2021). Stabilizing health care: an urgent call to action.

[33] Communications ZV. Zoom: One platform to connect. 5.12.2 ed: Zoom Video Communications; 2016.

[34] Weiner BJ, Lewis CC, Stanick C, Powell BJ, Dorsey CN, Clary AS (2017). Psychometric assessment of three newly developed implementation outcome measures. Implement Sci.

[35] Holt DT, Armenakis AA, Feild HS, Harris SG (2007). Readiness for organizational change: the systematic development of a scale. J Appl Behav Sci.

[36] Herscovitch L, Meyer JP (2002). Commitment to organizational change: extension of a three-component model. J Appl Psychol.

[37] Sekhon M, Cartwright M, Francis JJ (2017). Acceptability of healthcare interventions: an overview of reviews and development of a theoretical framework. BMC Health Serv Res.

[38] Murphy AL, Gardner DM (2019). Pharmacists' acceptability of a men's mental health promotion program using the theoretical framework of acceptability. AIMS Public Health.

[39] Corp. I (2017). SPSS statistics.

[40] Ferguson CJ (2009). An effect size primer: a guide for clinicians and researchers.

[41] Braun V, Clarke V (2006). Using thematic analysis in psychology. Qual Res Psychol.

[42] DeCuir-Gunby JT, Marshall PL, McCulloch AW (2011). Developing and using a codebook for the analysis of interview data: an example from a professional development research project. Field Methods.

[43] Craig P, Dieppe P, Macintyre S, Michie S, Nazareth I, Petticrew M (2008). Developing and evaluating complex interventions: the new medical research council guidance. BMJ.

[44] Haynes B (1999). Can it work? Does it work? Is it worth it? The testing of healthcareinterventions is evolving. BMJ.

[45] Klaic M, Kapp S, Hudson P, Chapman W, Denehy L, Story D (2022). Implementability of healthcare interventions: an overview of reviews and development of a conceptual framework. Implement Sci.

[46] Moore GF, Audrey S, Barker M, Bond L, Bonell C, Hardeman W (2015). Process evaluation of complex interventions: medical research council guidance. Br Med J.

[47] Vickers KS, Ridgeway JL, Hathaway JC, Egginton JS, Kaderlik AB, Katzelnick DJ (2013). Integration of mental health resources in a primary care setting leads to increased provider satisfaction and patient access. Gen Hosp Psychiatry.

[48] Bell L, Cornish P, Gauthier R, Kargus C, Rash J, Robbins R (2020). Implementation of the Ottawa hospital pain clinic stepped care program: a preliminary report. Can J Pain.

[49] Williams ME, Latta J, Conversano P (2008). Eliminating the wait for mental health services. J Behav Health Serv Res.

[50] Reichert A, Jacobs R (2018). The impact of waiting time on patient outcomes: evidence from early intervention in psychosis services in England. Health Econ.

[51] Windle E, Tee H, Sabitova A, Jovanovic N, Priebe S, Carr C (2020). Association of patient treatment preference with dropout and clinical outcomes in adult psychosocial mental health interventions: a systematic review and meta-analysis. JAMA Psychiat.

[52] Michie S, van Stralen MM, West R (2011). The behaviour change wheel: a new method for characterising and designing behaviour change interventions. Implement Sci.

[53] Powell BJ, Waltz TJ, Chinman MJ, Damschroder LJ, Smith JL, Matthieu MM (2015). A refined compilation of implementation strategies: results from the expert recommendations for implementing change (ERIC) project. Implement Sci.

[54] Waltz TJ, Powell BJ, Matthieu MM, Damschroder LJ, Chinman MJ, Smith JL (2015). Use of concept mapping to characterize relationships among implementation strategies and assess their feasibility and importance: results from the expert recommendations for implementing change (ERIC) study. Implement Sci.

[55] Damschroder LJ, Reardon CM, Widerquist MAO, Lowery J (2022). The updated consolidated framework for implementation research based on user feedback. Implement Sci.

[56] Carey RN, Connell LE, Johnston M, Rothman AJ, de Bruin M, Kelly MP (2019). Behavior change techniques and their mechanisms of action: a synthesis of links described in published intervention literature. Ann Behav Med.

[57] Waltz TJ, Powell BJ, Fernández ME, Abadie B, Damschroder LJ (2019). Choosing implementation strategies to address contextual barriers: diversity in recommendations and future directions. Implement Sci.

[58] Bower P, Kontopantelis E, Sutton A, Kendrick T, Richards DA, Gilbody S (2013). Influence of initial severity of depression on effectiveness of low intensity interventions: meta-analysis of individual patient data. BMJ.

[59] Chan SW, Adams M (2014). Service use, drop-out rate and clinical outcomes: a comparison between high and low intensity treatments in an IAPT service. Behav Cogn Psychother.

[60] Vaillancourt K, Manley J, McNulty N (2015). Why has our recovery rate dropped? An audit examining waiting times, starting scores and length of treatment in relation to recovery within an IAPT service. Cogn Behav Ther..

[61] Cremers G, Taylor E, Hodge L, Quigley A (2022). Effectiveness and acceptability of low-intensity psychological interventions on the well-being of older adults: a systematic review. Clin Gerontol.

[62] Richards DA, Bower P, Pagel C, Weaver A, Utley M, Cape J (2012). Delivering stepped care: an analysis of implementation in routine practice. Implement Sci.

[63] Shimokawa K, Lambert MJ, Smart DW (2010). Enhancing treatment outcome of patients at risk of treatment failure: meta-analytic and mega-analytic review of a psychotherapy quality assurance system. J Consult Clin Psychol.

[64] Woodward EN, Singh RS, Ndebele-Ngwenya P, Melgar Castillo A, Dickson KS, Kirchner JE (2021). A more practical guide to incorporating health equity domains in implementation determinant frameworks. Implement Sci Commun.

[65] Lambert MJ, Whipple JL, Hawkins EJ, Vermeersch DA, Nielsen SL, Smart DW (2003). Is it time for clinicians to routinely track patient outcome? A meta-analysis. Clin Psychol Sci Pract.

